# patRoon: open source software platform for environmental mass spectrometry based non-target screening

**DOI:** 10.1186/s13321-020-00477-w

**Published:** 2021-01-06

**Authors:** Rick Helmus, Thomas L. ter Laak, Annemarie P. van Wezel, Pim de Voogt, Emma L. Schymanski

**Affiliations:** 1 Institute for Biodiversity and Ecosystem Dynamics, University of Amsterdam, P.O. Box 94240, 1090 GE Amsterdam, The Netherlands; 2 KWR Water Research Institute, Chemical Water Quality and Health, P.O. Box 1072, 3430 BB Nieuwegein, The Netherlands; 3 Luxembourg Centre for Systems Biomedicine (LCSB), University of Luxembourg, L-4367 Belvaux, Luxembourg

**Keywords:** High resolution mass spectrometry, Compound identification, Non-target analysis, Computational workflows

## Abstract

Mass spectrometry based non-target analysis is increasingly adopted in environmental sciences to screen and identify numerous chemicals simultaneously in highly complex samples. However, current data processing software either lack functionality for environmental sciences, solve only part of the workflow, are not openly available and/or are restricted in input data formats. In this paper we present *patRoon*, a new *R* based open-source software platform, which provides comprehensive, fully tailored and straightforward non-target analysis workflows. This platform makes the use, evaluation and mixing of well-tested algorithms seamless by harmonizing various common (primarily open) software tools under a consistent interface. In addition, *patRoon* offers various functionality and strategies to simplify and perform automated processing of complex (environmental) data effectively. *patRoon* implements several effective optimization strategies to significantly reduce computational times. The ability of *patRoon* to perform time-efficient and automated non-target data annotation of environmental samples is demonstrated with a simple and reproducible workflow using open-access data of spiked samples from a drinking water treatment plant study. In addition, the ability to easily use, combine and evaluate different algorithms was demonstrated for three commonly used feature finding algorithms. This article, combined with already published works, demonstrate that *patRoon* helps make comprehensive (environmental) non-target analysis readily accessible to a wider community of researchers.

## Introduction

Chemical analysis is widely applied in environmental sciences such as earth sciences, biology, ecology and environmental chemistry, to study, e.g. geomorphic processes (chemical) interaction between species or the occurrence, fate and effect of chemicals of emerging concern in the environment. The environmental compartments investigated include air, water, soil, sediment and biota, and exhibit a highly diverse chemical composition and complexity. The number and quantities of chemicals encountered within samples may span several orders of magnitude relative to each other. Therefore, chemical analysis must discern compounds at ultra-trace levels, a requirement that can be largely met with modern analytical instrumentation such as liquid or gas chromatography coupled with mass spectrometry (LC-MS and GC–MS). The high sensitivity and selectivity of these techniques enable accurate identification and quantification of chemicals in complex sample materials.

Traditionally, a ‘target analysis’ approach is performed, where identification and quantitation occur by comparing experimental data with reference standards. The need to pre-select compounds of interest constrains the chemical scope of target analysis, and hampers the analysis of chemicals with (partially) unknown identities such as transformation products and contaminants of emerging concern (CECs). In addition, the need to acquire or synthesize a large number of analytical standards may not be feasible for compounds with a merely suspected presence. Recent technological advancements in chromatography and high resolution MS (HRMS) allows detection and tentative identification of compounds without the prior need of standards [1]. This ‘non-target’ analysis (NTA) approach is increasingly adopted to perform simultaneous screening of up to thousands of chemicals in the environment, such as finding new CECs [1–6], identifying chemical transformation (by)products [7–12] and identification of toxicants in the environment [13–16].

Studies employing environmental NTA typically allow the detection of hundreds to thousands of different chemicals [17, 18]. Effectively processing such data requires workflows to automatically extract and prioritize NTA data, perform chemical identification and assist in interpreting the complex resulting datasets. Currently available tools often originate from other research domains such as life sciences and may lack functionality or require extensive optimization before being suitable for environmental analysis. Examples include handling chemicals with low sample-to-sample abundance, recognition of halogenated compounds, usage of data sources with environmentally relevant substances, or temporal and spatial trends [1, 2, 5, 6, 9, 19].

**Fig. 1:**
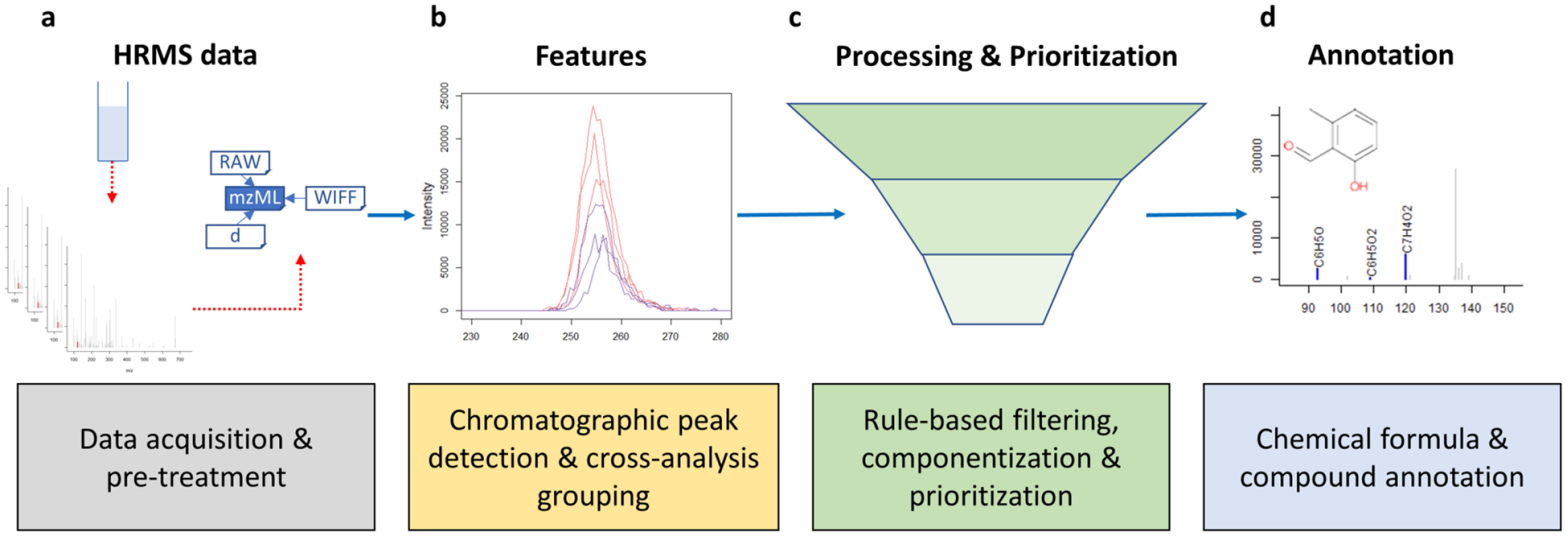
Generic workflow for environmental non-target analysis

An NTA workflow can be generalized as a four step process (Fig. 1) [1]. Firstly, data from LC or GC-HRMS is either acquired or retrieved retrospectively, and pre-treated for subsequent analysis (Fig. 1a). This pre-treatment may involve conversion to open data formats (e.g. mzML [20] or mzXML [21]) to increase operability with open-source software, re-calibration of mass spectra to improve accuracy and centroiding [22] or other raw data reduction steps to conserve space such as trimming chromatographs or filtering mass scans (e.g. with the functionality from the ProteoWizard suite [23]). Secondly (Fig. 1b), features with unique chromatographic and mass spectral properties (e.g. retention time, accurate mass, signal intensity) are automatically extracted and features considered equivalent across sample analyses are grouped to allow qualitative and (semi-) quantitative comparison further down the workflow. Thirdly (Fig. 1c), the feature dataset quality is refined, for instance, via rule-based filters (e.g. minimum intensity and absence in sample blanks) and grouping of features based on a defined relationship such as adducts or homologous series (e.g. “componentization”). Further prioritization during this step of the workflow is often required for efficient data analysis, for instance, based on chemical properties (e.g. mass defect and isotopic pattern), suspected presence (i.e. “suspect screening”) or intensity trends in time and/or space (e.g. reviewed in [1]). Finally (Fig. 1d), prioritized features are annotated, for instance by assigning chemical formulae or compounds from a chemical database (e.g. *PubChem* [24] or *CompTox* [25]) based on the exact mass of the feature. The resulting candidates are ranked by conformity with MS data, such as match with theoretical isotopic pattern and in silico or library MS fragmentation spectra, and study-specific metadata, such as number of scientific references and toxicity data [1, 19].

Various open and closed software tools are already available to implement (parts of) the NTA workflow. Commercial software tools such as *MetaboScape* [26], *UNIFI* [27], *Compound Discoverer* [28] and *ProGenesis QI* [29] provide a familiar and easy to use graphical user interface, may contain instrument specific functionality and optimizations and typically come with support for their installation and usage. However, they are generally not open-source or open-access and are often restricted to proprietary and specific vendor data formats. This leads to difficulties in data sharing, as exact algorithm implementations and parameter choices are hidden, while maintenance, auditing or code extension by other parties is often not possible. Many open-source or open-access tools are available to process mass spectrometry data, such as *CFM*-*ID* [30, 31], *enviMass* [32], *enviPick* [33], *nontarget* [34], *GenForm* [35], *MetFrag* [36], *FOR*-*IDENT* [37], *MS*-*DIAL* [38], *MS*-*FINDER* [39], *MZmine* [40], *OpenMS* [41], *ProteoWizard* [23], *RAMClustR* [42], *SIRIUS* and *CSI:FingerID* [43–47], *XCMS* [48], *CAMERA* [49] and *XCMS online* [50] (Table 1, further reviewed in [51, 52]). Various open tools are easily interfaced with the *R* statistical environment [53] (Table 1). Leveraging this open scripting environment inherently allows defining highly flexible and reproducible workflows and increases the accessibility of such workflows to a wider audience as a result of the widespread usage of *R* in data sciences. While many tools were originally developed to process metabolomics and proteomics data, approaches such as *XCMS* and *MZmine* have also been applied to environmental NTA studies [6, 54]. However, as stated above, these tools can lack the specific functionality and optimizations required for effective environmental NTA data processing. While a complete environmental NTA workflow requires several steps from data pre-processing through to automated annotation (see Fig. 1), existing software approaches designed for processing environmental data (e.g. *enviMass* and *nontarget*) and most others only implement part of the required functionality, as indicated in Table 1. Furthermore, only few workflow solutions support automated compound annotation. Moreover, available tools often overlap in functionality (Table 1), and are implemented with differing algorithms or employing different data sources. Consequently, tools may generate different results, as has been shown when generating feature data [55–59] or performing structural annotations [19, 60]. Hence, the need to learn, combine, optimize and sometimes develop or adapt various specialized software tools, and perform tedious transformation of datasets currently hinders further adoption of NTA, especially in more routine settings lacking appropriate in-house computational expertise. Thus, before NTA is fully “ready to go” [1], a new platform is necessary that (a) is independent of closed MS vendor input data, (b) incorporates optimizations and functionality necessary for a complete environmental NTA workflow and (c) allows researchers to seamlessly combine and evaluate existing and well-tested algorithms in order to tailor an optimal NTA workflow to the particular study types and methodological characteristics.

**Table 1:** Overview of commonly used open-source or open-access software tools to implement NTA workflows

		HRMS	Features					Annotation								Interface	Language	OS	License	References
		Pre-process	Find	Group^1^	Clean-up	Suspects		MS extr^2^	Formula	Comp pred^3^	Comp lib^3^	Hom extr^4^	Group^5^	Clean-up	RT pred^6^					
a	*CFM*-*ID*									X	X					CLI, Web	C++	Cross	LGPLv2.1	[30, 31]
b	*enviMass, enviPick, nontarget*	X^i^	*X*	X	X	X						*X*	X			GUI, R, Web	R	Cross	GPLv3.0^7^	[32–34]
c	*GenForm*								*X*							CLI	C++	Cross^8^	LGPLv2.0	[35]
d	*MetFrag*									*X*	*X*			X	X	CLI, R, Web	Java	Cross	LGPLv2.0	[36]
e	*FOR*-*IDENT*									X^d^	X				X	Web	HTML	Cross	Closed	[37]
f	*MS*-*DIAL,* *MS*-*FINDER*		X	X	X	X		X	X	X	X		X			CLI, GUI	C#	Win	LGPLv3.0	[38, 39]
g	*MZmine*	X	X^gl^	X	X	X		X	X	X^k^	X		X^gl^			GUI	Java	Cross	GPLv2.0	[40]
h	*OpenMS*	*X*^hi^	*X*	*X*				X		X^k^	X		X			CLI, GUI, Python	C++	Win, Lin, Mac	BSD/3-Clause	[41]
i	*ProteoWizard*	*X*														CLI, GUI	C++	Win, Lin	Apache 2.0	[23]
j	*RAMClustR*							X					*X*			R	R	Cross	GPLv2.0	[42]
k	*SIRIUS and CSI:FingerID*								*X*	*X*				X		CLI, GUI	Java	Cross	GPLv3.0	[43–47]
l	*XCMS and CAMERA*		*X*	*X*	X								*X*			R	R	Cross	GPLv2.0	[48, 49]
m	*XCMS Online*	X	X^l^	X^l^				X			X		X			Web	R	Cross	Closed	[50]
n	*patRoon*	*X*^hi^	*X*^bhl^	*X*^hl^	*X*	*X*		*X*	*X*^ck^	*X*^dk^	*X*^d^	*X*^b^	*X*^jl^	*X*	*X*^d^	R	R	Cross	GPLv3.0	

(1): Group features across samples; (2): automatic MS data extraction for annotation purposes; (3): Compound annotation (in silico/library); (4): unsupervised homologous series extraction; (5): grouping and annotating chemically related features (e.g. adducts, isotopes, in-source fragments); (6): retention time prediction; (7): enviMass is distributed commercially; (8): Only *Microsoft Windows* binaries are distributed

*CLI* command-line interface, *GUI* graphical user interface, *Web* interfaced via internet browser, *OS* supported operating systems, *Win* Microsoft Windows, *Lin* GNU/Linux, *Mac* macOS, *Cross* cross-platform

Italic: functionality integrated in *patRoon*

Superscript: implemented with algorithms by given rows (omitted if only native)

Here, we present an *R* based open-source software platform called *patRoon* (‘pattern’ in Dutch) providing comprehensive NTA data processing from HRMS data pre-treatment, detection and grouping of features, through to molecular formula and compound annotation. This is achieved by harmonizing various commonly used (and primarily open) tools in a consistent and easy to use interface, which provides access to well-established algorithms without aforementioned limitations when used alone. Complementary and novel functionality is implemented, such as automated chemical annotation, visualization and reporting of results, comparing and combining results from different algorithms, and data reduction and prioritization strategies, which further improve and simplify effective NTA data processing. The architecture of *patRoon* is designed to be extendable in order to accommodate for rapid developments in the NTA research field.

### Implementation

The implementation section starts with an overview of the *patRoon* workflows. Subsequent sections provide details on novel functionality implemented by *patRoon,* which relate to data processing, annotation, visualization and reporting. Finally, a detailed description is given of the software architecture. *patRoon* is then demonstrated in the Results and discussion section. The software tools and databases used for the implementation of *patRoon* are summarized in Additional file 1.

#### Workflow in patRoon

**Fig. 2:**
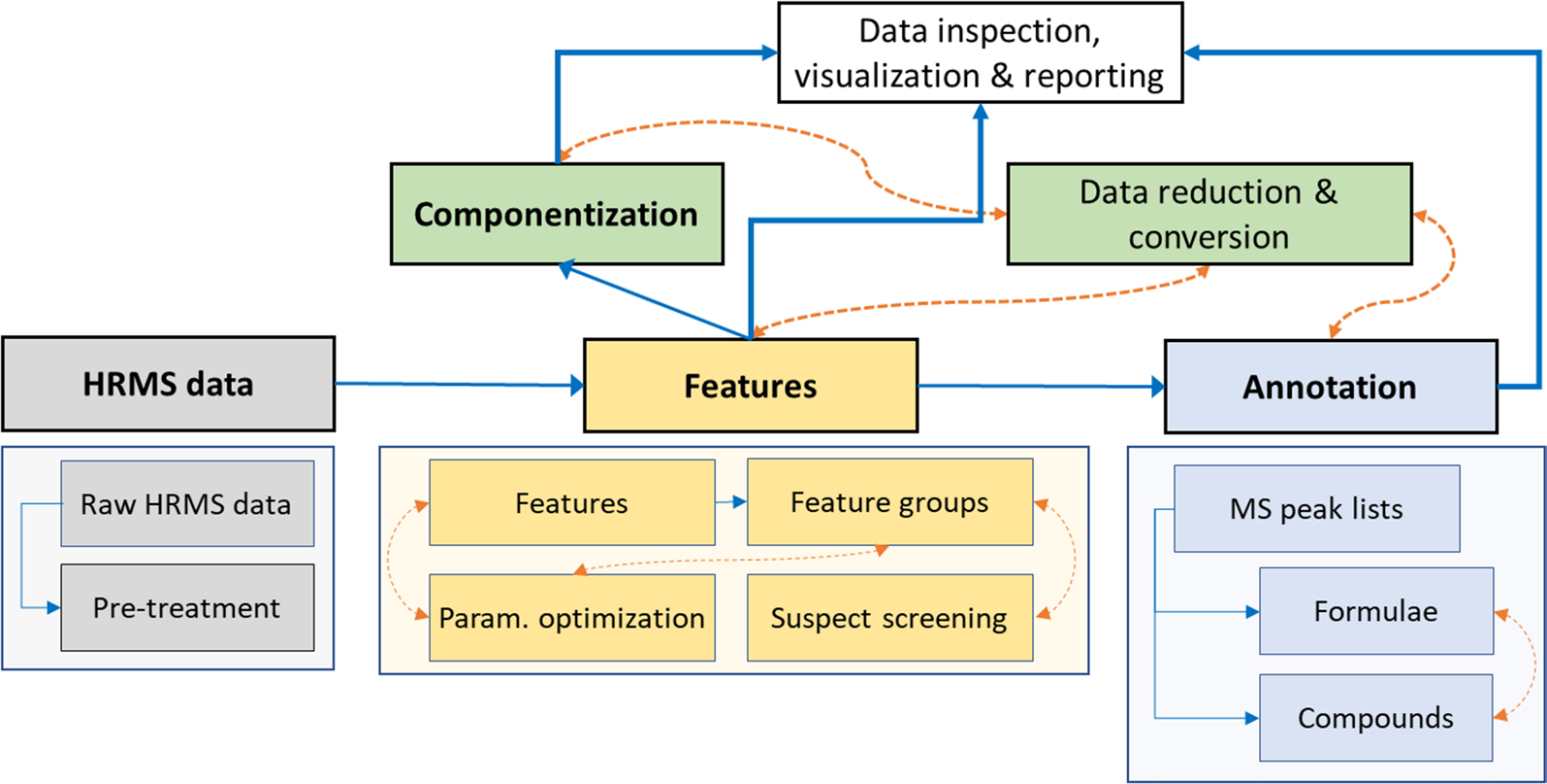
Overview of the NTA *patRoon* workflow. All steps are optional. Steps that are connected by blue and straight arrows represent a one-way data dependency, whereas steps connected with red curved and dashed arrows represent steps with two-way data interaction

*patRoon* encompasses a comprehensive workflow for HRMS based NTA (Fig. 2). All steps within the workflow are optional and the order of execution is largely customizable. Some steps depend on data from previous steps (blue arrows) or may alter or amend data from each other (red arrows). The workflow commonly starts with pre-treatment of raw HRMS data. Next, feature data is generated, which consists of finding features in each sample, an optional retention time alignment step, and then grouping into “feature groups”. Finding and grouping of features may be preceded by automatic parameter optimization, or followed by suspect screening. The feature data may then finally be used for componentization and/or annotation steps, which involves generation of MS peak lists, as well as formula and compound annotations. At any moment during the workflow, the generated data may be inspected, visualized and treated by, e.g. rule based filtering. These operations are discussed in the next section.

Several commonly used open software tools, such as *ProteoWizard* [23], *OpenMS* [41], *XCMS* [48], *MetFrag* [36] and *SIRIUS* [43–47], and closed software tools, such as *Bruker DataAnalysis* [61] (chosen due to institutional needs), are interfaced to provide a choice between multiple algorithms for each workflow step (Additional file 3: Table S1). Customization of the NTA workflow may be achieved by freely selecting and mixing algorithms from different software tools. For instance, a workflow that uses *XCMS* to group features allows that these features originate from other algorithms such as *OpenMS*, a situation that would require tedious data transformation when *XCMS* is used alone. Furthermore, the interface with tools such as *ProteoWizard* and *DataAnalysis* provides support to handle raw input data from all major MS instrument vendors.

To ease parameter selection over the various feature finding and grouping algorithms, an automated feature optimization approach was adopted from the isotopologue parameter optimization (*IPO*) *R* package [62], which employs design of experiments to optimize LC–MS data processing parameters [63]. IPO was integrated in *patRoon*, and its code base was extended to (a) support additional feature finding and grouping algorithms from *OpenMS*, *enviPick* and usage of the new *XCMS 3* interface, (b) support isotope detection with *OpenMS*, (c) perform optimization of qualitative parameters and (d) provide a consistent output format for easy inspection and visualization of optimization results.

In *patRoon*, componentization refers to consolidating different (grouped) features with a prescribed relationship, which is currently either based on (a) highly similar elution profiles (i.e. retention time and peak shape), which are hypothesized to originate from the same chemical compound (based on [42, 49]), (b) participation in the same homologous series (based on [64]) or (c) the intensity profiles across samples (based on [4, 5, 65]). Components obtained by approach (a) typically comprise adducts, isotopologues and in-source fragments, and these are recognized and annotated with algorithms from CAMERA [49] or RAMClustR [42]. Approach (b) uses the *nontarget R* package [34] to calculate series from aggregated feature data from replicates. The interpretation of homologous series between replicates is assisted by merging series with overlapping features in cases where this will not yield ambiguities to other series. If merging would cause ambiguities, instead links are created that can then be explored interactively and visualized by a network graph generated using the *igraph* [66] and *visNetwork* [67] *R* packages (see Additional file 2: Figure S1).

During the annotation step, molecular formulae and/or chemical compounds are automatically assigned and ranked for all features or feature groups. The required MS peak list input data are extracted from all MS analysis data files and subsequently pre-processed, for instance, by averaging multiple spectra within the elution profile of the feature and by removing mass peaks below user-defined thresholds. All compound databases and ranking mechanisms supported by the underlying algorithms are supported by *patRoon* and can be fully configured. Afterwards, formula and structural annotation data may be combined to improve candidate ranking and manual interpretation of annotated spectra. More details are outlined in the section “MS peak list retrieval, annotation and candidate ranking”.

#### Data reduction, comparison and conversion

Various rule-based filters are available for data-cleanup or study specific prioritization of all data obtained through the workflow (see Table 2), and can be inverted to inspect the data that would be removed (i.e. negation). To process feature data, multiple filters are often applied, however, the order may influence the final result. For instance, when features were first removed from blanks by an intensity filter, a subsequent blank filter will not properly remove these features in actual samples. Similarly, a filter may need a re-run after another to ensure complete data clean-up. To reduce the influence of order upon results, filters for feature data are executed by default as follows:

1. An intensity pre-filter, to ensure good quality feature data for subsequent filters;
2. Filters not affected by other filters, such as retention time and *m/z* range;
3. Minimum replicate abundance, blank presence and ‘regular’ minimum intensity;
4. Repetition of the replicate abundance filter (only if previous filters affected results);
5. Other filters that are possibly influenced by prior steps, such as minimum abundance in feature groups or sample analyses.

**Table 2:** Major rule-based filtering functionality implemented in patRoon

Filter functionality	Features	Feature groups	MS peak lists	Formulae	Compounds	Components
Intensity threshold	X	X	X			
Feature properties^a^	X	X				
Max intensity deviation across replicates		X				
Minimum intensity above blank		X				
Minimum size or abundance		X				X
Top most abundant/highest scoring			X	X	X	
Minimum scoring				X	X	
Annotation^b^				X	X	X
Organic matter rules^c^				X		

^a^Retention time, chromatographic peak width, m/z and mass defect range

^b^e.g. adducts, isotopologues, formula composition, neutral loss

^c^expected formula composition based on [68–71]

Note that the above scheme only applies to those filters requested by the user, and the user can apply another order if desired.

Further data subsetting allows the user to freely select data of interest, for instance, following a (statistical) prioritization approach performed by other tools. Similarly, features that are unique or overlapping in different sample analyses may be isolated, which is a straightforward but common prioritization technique for NTA studies that involve the comparison of different types of samples.

Data from feature groups, components or annotations that are generated with different algorithms (or parameters thereof) can be compared to generate a consensus by only retaining data with (a) minimum overlap, (b) uniqueness or (c) by combining all results (only (c) is supported for data from components). Consensus data are useful to remove outliers, for inspection of algorithmic differences or for obtaining the maximum amount of data generated during the workflow. The consensus for formula and compound annotation data are generated by comparison of Hill-sorted formulae and the skeleton layer (first block) of the InChIKey chemical identifiers [72], respectively. For feature groups, where different algorithms may output deviating retention and/or mass properties, such a direct comparison is impossible. Instead, the dimensionality of feature groups is first reduced by averaging all feature data (i.e. retention times, *m/z* values and intensities) for each group. The collapsed groups have a similar data format as ‘regular’ features, where the compared objects represent the ‘sample analyses’. Subjection of this data to a feature grouping algorithm supported by *patRoon* (i.e. from *XCMS* or *OpenMS*) then allows straightforward and reliable comparison of feature data from different algorithms, which is finally used to generate the consensus.

Hierarchical clustering is utilized for componentization of features with similar intensity profiles or to group chemically similar candidate structures of an annotated feature. The latter “compound clustering” assists the interpretation of features with large numbers of candidate structures (e.g. hundreds to thousands). This method utilizes chemical fingerprinting and chemical similarity methods from the *rcdk* package [73] to cluster similar structures, and subsequent visual inspection of the maximum common substructure then allows assessment of common structural properties among candidates (methodology based on [74]). Cluster assignment for both componentization and compound annotation approaches is performed automatically using the *dynamicTreeCut R* package [75]. However, clusters may be re-assigned manually by the desired amount or tree height.

Several data conversion methods were implemented to allow interoperability with other software tools. All workflow data types are easily converted to commonly used *R* data types (e.g. data.frame or list), which allows further processing with other *R* packages. Furthermore, feature data may be converted to and from native *XCMS* objects (i.e. xcmsSet and XCMSnExp) or exported to comma-separated values (CSV) formats compatible with *Bruker ProfileAnalysis* or *TASQ*, or *MZmine*.

#### MS peak list retrieval, annotation and candidate ranking

Data for MS and MS/MS peak lists for a feature are collected from spectra recorded within the chromatographic peak and averaged to improve mass accuracies and signal to noise ratios. Next, peak lists for each feature group are assigned by averaging the mass and intensity values from peak lists of the features in the group. Mass spectral averaging can be customized via several data clean-up filters and a choice between different mass clustering approaches, which allow a trade-off between computational speed and clustering accuracy. By default, peak lists for MS/MS data are obtained from spectra that originate from precursor masses within a certain tolerance of the feature mass. This tolerance in mass search range is configurable to accommodate the precursor isolation window applied during data acquisition. In addition, the precursor mass filter can be completely disabled to accommodate data processing from data-independent MS/MS experiments, where all precursor ions are fragmented simultaneously.

The formula annotation process is configurable to allow a tradeoff between accuracy and calculation speeds. Candidates are assigned to each feature group, either directly by using group averaged MS peak list data, or by a consensus from formula assignments to each individual feature in the group. While the latter inherently consumes more time, it allows removal of outlier candidates (e.g. false positives due to features with poor spectra). Candidate ranking is improved by inclusion of MS/MS data in formula calculation (optional for *GenForm* [35] and *DataAnalysis*).

Formula calculation with *GenForm* ranks formula candidates on isotopic match (amongst others), where any other mass peaks will penalize scores. Since MS data of “real-world” samples typically includes many other mass peaks (e.g. adducts, co-eluting features, background ions), *patRoon* improves the scoring accuracy by automatic isolation of the feature isotopic clusters prior to *GenForm* execution. A generic isolation algorithm was developed, which makes no assumptions on elemental formula compositions and ion charges, by applying various rules to isolate mass peaks that are likely part of the feature isotopic cluster (see Additional file 2: Figure S2). These rules are configured to accommodate various data and study types by default. Optimization is possible, for instance, to (a) improve studies of natural or anthropogenic compounds by lowering or increasing mass defect tolerances, respectively, (b) constrain cluster size and intensity ranges for low molecular weight compounds or (c) adjust to expected instrumental performance such as mass accuracy. Note that precursor isolation can be performed independently of formula calculation, which may be useful for manual inspection of MS data.

Compound annotation is usually the most time and resource intensive process during the non-target workflow. As such, instead of annotating individual features, compound assignment occurs for the complete feature group. All compound databases supported by the underlying algorithms, such as *PubChem* [24], *ChemSpider* [76] or *CompTox* [25] and other local CSV files, as well as the scoring terms present in these databases, such as in silico and spectral library MS/MS match, references in literature and presence in suspect lists, can be utilized with *patRoon*. Default scorings supported by the selected algorithm/database or sets thereof are easily selectable to simplify effective compound ranking. Furthermore, formula annotation data may be incorporated in compound ranking, where a ‘formula score’ is calculated for each candidate formula, which is proportional to its ranking in the formula annotation data. Execution of unattended sessions is assisted by automatic restarts after occurrence of timeouts or errors (e.g. due to network connectivity) and automatic logging facilities.

#### Visualization, reporting and graphical interface

In *patRoon*, visualization functionality is provided for feature and annotation data (e.g. extracted ion chromatograms (EICs) and annotated spectra), to compare workflow data (i.e. by means of Venn, chord and UpSet [77] diagrams, using the *VennDiagram* [78], *circlize* [79] and *UpSetR* [80] *R* packages, respectively) and others such as plotting results from automatic feature optimization experiments and hierarchical clustering data. Reports can be generated in a common CSV text format or in a graphical format via export to a portable document file (PDF) or hypertext markup language (HTML) format. The latter are generated with the *R Markdown* [81, 82] and *flexdashboard* [83] *R* packages, and provide an easy to use interface for interactive sorting, searching and browsing reported data. As plotting and reporting functionalities can be performed at any stage during the workflow, the data that is included in the reports is fully configurable.

**Fig. 3:**
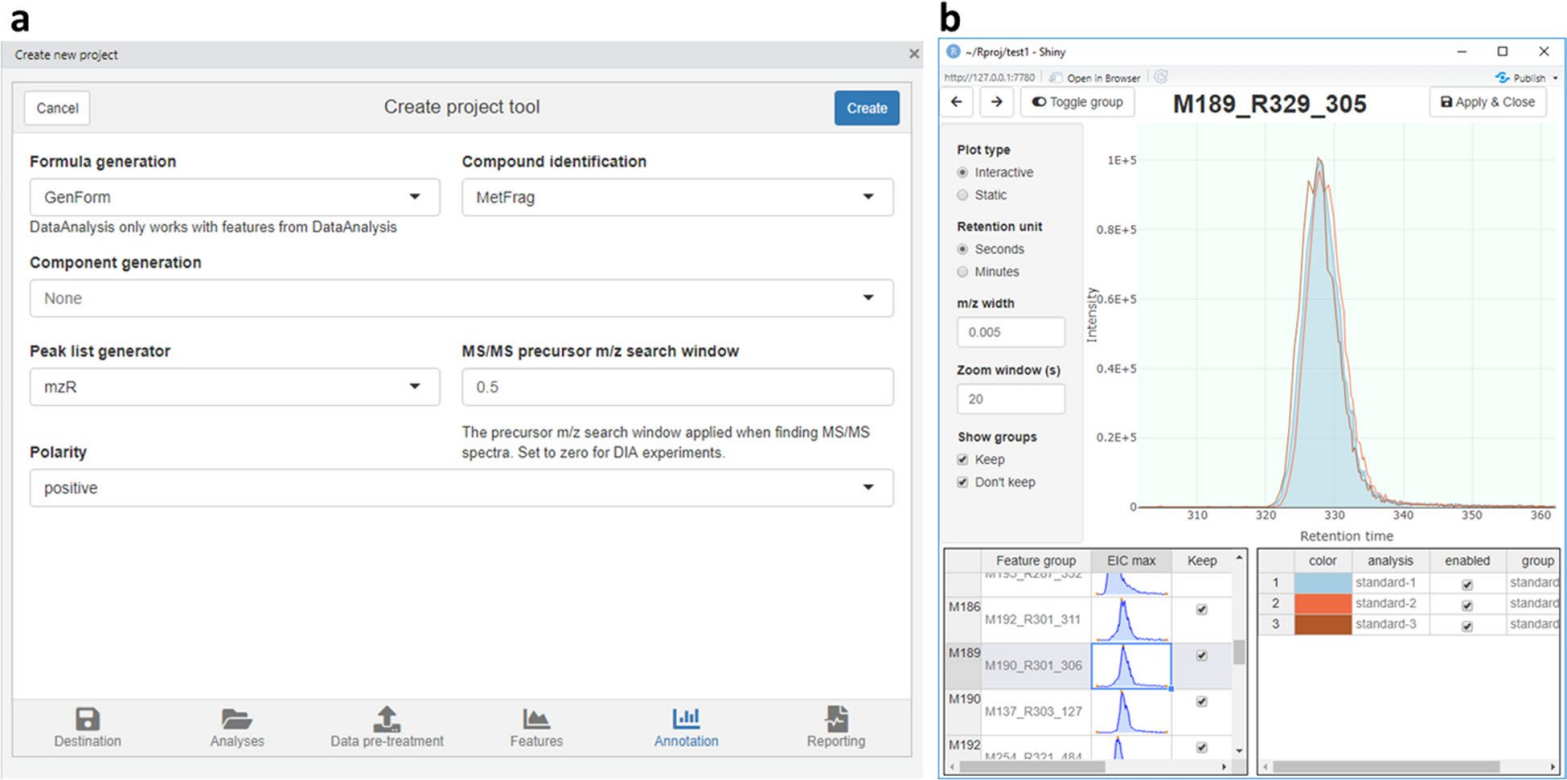
Graphical user interface tools in *patRoon*. Tools are provided **a** to create a new *patRoon* data analysis project and **b** to inspect feature chromatography data

While *patRoon* is primarily interfaced through *R*, several graphical user interface tools are provided to assist the (novice) user. Most importantly, *patRoon* provides a *Shiny* [84] based graphical user interface tool that automatically generates a commented template *R* script from visual user parameter input selection, such as MS data input files, workflow algorithms and other common workflow parameters (Fig. 3a). Secondly, chromatographic data of features may be inspected either by automatic addition of EICs in a *Bruker DataAnalysis* session or with a *Shiny* graphical based interface (Fig. 3b).

#### Software architecture

*patRoon* is distributed as an *R* package. Its source code is primarily written in the *R* language, with some support code written in C++ and JavaScript. Both *Microsoft Windows* (hereafter referred to as *Windows*) and *Linux* platforms are supported (support for macOS is envisaged in the future). Several external dependencies are required; notable examples are in Additional file 3: Table S1. *GenForm* is automatically compiled during package installation. For *Windows* platforms, an installation script is provided to install and configure *patRoon* and all of its dependencies automatically. Documentation includes a handbook, tutorial and full reference manual [85–88], which are produced with the *bookdown* [89, 90], *R Markdown* and *roxygen2* [91] *R* packages, respectively. Example data is contained in the *patRoonData R* package [92, 93].

**Fig. 4:**
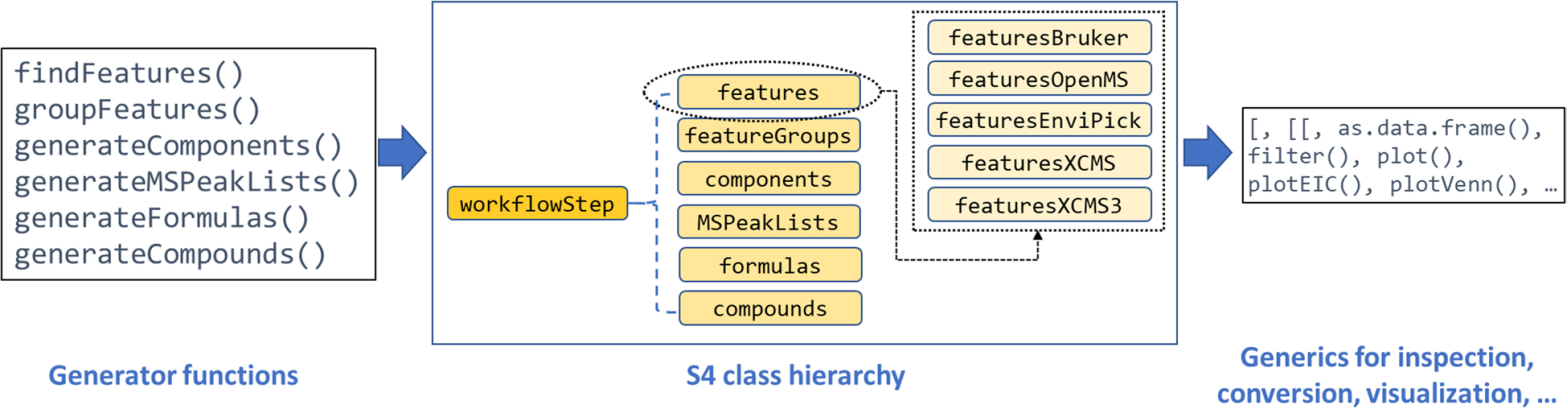
Interface for the *patRoon* workflow. The workflow steps are performed by a set of functions that execute the selected algorithm and return the data in a harmonized format by utilizing the ‘S4’ object oriented programming approach of *R*. These objects all derive from a common base class and may be further sub-classed in algorithm specific classes (as is exemplified for features). Generic functions are defined for all workflow classes to implement further data processing functionality in a predictable and algorithm independent manner (see also Table 3). Further information is provided in the reference manual [85, 86]

An important design goal was to provide a consistent, generic and easy to use interface that does not require the user to know the implementation and interfacing details of the supported algorithms. Each workflow step is executed by a generator function that takes the desired algorithm and its parameters as input and returns objects from a common set of data formats (see Fig. 4). Names for commonly used parameters supported by multiple algorithms are standardized for consistency and defaults are set where reasonable. Furthermore, the format of input data such as retention time units as well as formula and adduct specifications are harmonized and automatically converted to the format expected by the algorithm. Nearly all parameters from the underlying algorithm can be set by the user, hence, full configurability of the workflow is retained wherever possible. Generic naming schemes are applied to output data, which assist the user in comparing results originating from different algorithms. All exported functions from *patRoon* verify user input with the *checkmate* [94] package, which efficiently performs tests such as correctness of value range and type, and prints descriptive messages if input is incorrect.

A set of generic methods are defined for workflow classes that perform general data inspection, selection, conversion and visualization, irrespective of the algorithm that was used to generate the object (see Table 3). Consequently, the implementation of common function names for multiple output classes allows a predictable and consistent user interface.

**Table 3:** Common generic methods defined in patRoon to process workflow data

Generic	Purpose
length(), show(), algorithm(), names(), groupNames()	Obtain general object information such as object length and unique identifiers for contained results
filter()	Rule-based filtering operations
[, [[, $ operators	Subsetting or extracting data
as.data.table(), as.data.frame()	Conversion to data.table or data.frame object
unique(), overlap()	Extract unique or overlapping features across replicates
consensus()	Generates a consensus between different objects of the same class
plot(), plotEIC(), plotSpec()	Plot general, chromatographic and annotation data
plotChord(), plotUpSet(), plotVenn()	Comparison of feature data or workflow objects from different algorithms by chord, UpSet and Venn diagrams

Several optimization strategies are employed in *patRoon* to reduce computational requirements and times. Firstly, external command line (CLI) tools are executed in parallel to reduce overall execution times for repetitive (e.g. per sample analysis or per feature) calculations. Commands are queued (first in, first out) and their execution is handled with the *processx* package [95]. Secondly, functions employing time intensive algorithms automatically cache their (partial) results in a local *SQLite* database file, which is accessed via the *DBI* [96] and *RSQLite* [97] *R* packages. Thirdly, performance critical code dealing with *OpenMS* data files and loading chromatographic data was written in C++ (interfaced with *Rcpp* [98–100]) to significantly reduce times needed to read or write data. Fourthly, the output files from *OpenMS* tools are loaded in chunks using the *pugixml* software library [101] to ensure a low memory footprint. Finally, reading, writing and processing (large) internal tabular data is performed with the *data.table R* package, which is a generally faster and more memory efficient drop-in replacement to the native tabular data format of *R* (data.frame), especially for large datasets [102].

Interfacing with *ProteoWizard* [23], *OpenMS*, *GenForm*, *SIRIUS* and *MetFrag* occurs by wrapper code that automatically executes the CLI tools and perform the data conversions necessary for input and output files. An alternative interface to *MetFrag* is also provided by employing the *metfRag R* package [103], however, in our experience this option is currently significantly slower than the CLI and therefore not used by default. For tools that are not readily controllable from *R* (i.e. *ProfileAnalysis*, *TASQ* and *MZmine*), interfacing occurs via importing or exporting CSV files (only export is supported for *MZmine*). Finally, the *RDCOMClient R* package [104] is used to interface with *Bruker DataAnalysis* via the distributed component object model, which allows automation of *DataAnalysis* functionality from *R* that otherwise would only be available via its integrated visual basic scripting environment.

A continuous integration pipeline performs automated tests during development and delivers files to simplify installation of *patRoon* and all its dependencies (Additional file 2: Figure S3). More than 900 unit tests are performed (> 80% code coverage) with the *testthat* and *vdiffr R* packages [105, 106]. After successful test completion, the final step involves building (a) *Windows* binary *R* packages of *patRoon* and its dependencies and (b) *Linux* Docker images with a complete working environment of *patRoon* and the *RStudio* integrated development environment [107] (based on [108]), which both facilitate installation of *patRoon* with tested and compatible dependencies.

## Results and discussion

This section starts with benchmarks of important optimization strategies implemented in *patRoon*, and concludes with demonstrations on how *patRoon* can implement a common NTA workflow and the algorithm consensus functionality. Since the implementation of individual workflow steps, such as obtaining feature data and annotations, heavily rely on well-established algorithms that have been evaluated elsewhere, further evaluations have not been performed here. Furthermore, an objective comparison of *patRoon* with other NTA workflows is currently being performed as part of a collaborative trial organized by the NORMAN Network [109]. Recent applications of complete environmental NTA studies performed with *patRoon* are already described in several publications [7, 12, 14, 71, 110].

### Benchmark and demonstration data

The data used to benchmark and demonstrate *patRoon* were obtained with an LC-HRMS analysis of influent and effluent samples from two drinking water treatment pilot installations and a procedural blank. The pilot installations were fed by surface water (Meuse and IJsselmeer, the Netherlands) that were subjected to various pre-treatment steps (e.g. rapid and slow sand filtration, drum sieves and dune filtration). Effluent samples investigated in this study were produced after advanced oxidation utilizing O_3_ and H_2_O_2_ or ultrafiltration and reverse osmosis. Sample blanks were obtained from tap water. All samples were filtered in triplicate by 0.2 µm regenerated-cellulose filters. Influent samples were spiked with a set of 18 common environmental contaminants (see Table 5). The analyses were performed using an LC-HRMS Orbitrap Fusion system (ThermoFisher Scientific, Bremen, Germany) operating with positive electrospray ionization. Further details of the pilot installations and analytical conditions are described in [11]. The raw data files can be obtained from [111].

### Parallelization benchmarks

**Fig. 5:**
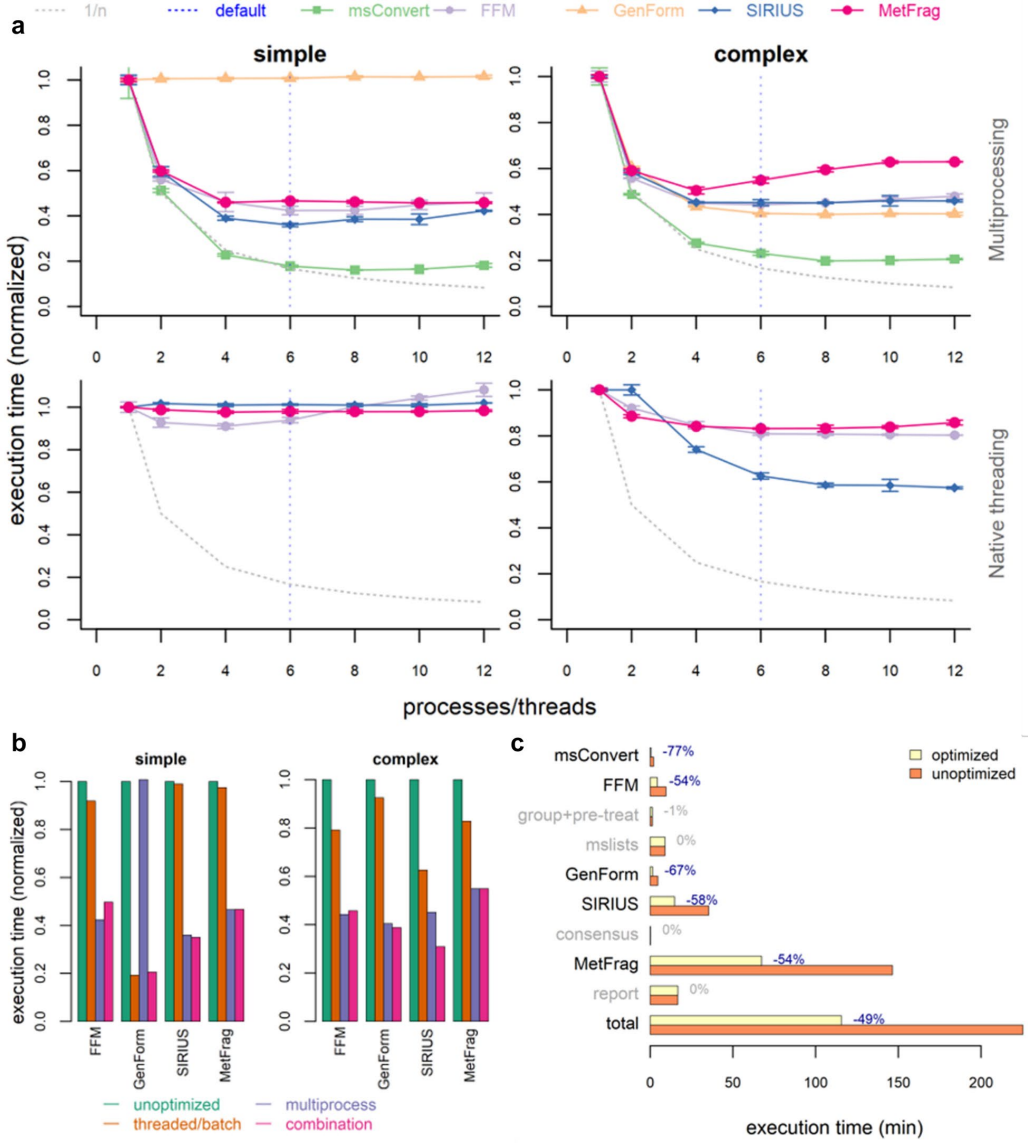
Parallelization benchmark results. **a** Benchmark results for commonly used CLI tools applied in *patRoon* workflows under varying parallelization conditions. The tested tools were *msConvert*, *FeatureFinderMetabo* (FFM), *GenForm*, *SIRIUS* and *MetFrag*. Tests were performed with “simple” (left) and “complex” (right) input conditions (Table 4) to simulate varying workflow complexity. Parallelization was performed with the multiprocessing functionality of *patRoon* (top) or by using native multithreading (bottom, for tools that supported this). Graphs represent number of processes or threads versus relative execution time (normalized to sequential results). The dotted grey lines represent the theoretical trend if maximum parallelization performance is achieved. The dashed blue line represents the number of physical cores that became the default selection in *patRoon* based on these results. **b** Comparison of execution times (normalized to the execution times of the unoptimized results) when tools are executed without optimizations (green), executed with native multithreading (*FeatureFinderMetabo*, *SIRIUS* and *MetFrag*) or batch mode (*GenForm*) (orange), executed with multiprocessing (purple) or a combination of the latter two (pink), using simple (left) and complex (right) input conditions. **c** Overview of execution times for a complete *patRoon* workflow executed under optimized versus unoptimized conditions. All results for *msConvert* and *SIRIUS* were obtained without enabling their native batch mode

Several benchmarks were performed to test the multiprocessing functionality of *patRoon*. Tests were performed on a personal computer equipped with an Intel^®^ Core™ i7-8700 K CPU (6 cores, 12 threads), 32 gigabyte RAM, SATA SSD storage and the *Windows* 10 Enterprise operating system. Benchmarks were performed in triplicate using the *microbenchmark R* package [112]. Standard deviations were below ten percent (see Fig. 5a). Benchmarking was performed on *msConvert*, *FeatureFinderMetabo*, *GenForm*, *SIRIUS* and *MetFrag*. The multiprocessing functionality was compared to native multithreading for the tools that supported this (*FeatureFinderMetabo*, *SIRIUS* and *MetFrag*). In addition, the performance of batch calculations with multiprocessing was compared with native batch calculation modes of tools where possible (*msConvert* and *SIRIUS*). Parallelization methods were tested with 1-12 parallel processes or threads (i.e. up to full utilization of both CPU threads of each core). Input conditions were chosen to simulate “simple” and “complex” workflows, where the latter resulted in more demanding calculations with ~ 2–10 × longer mean execution times (Table 4). The caching functionality of *patRoon* was disabled, where appropriate, to obtain representative and reproducible test results. Prior to benchmarking, candidate chemical compounds from *PubChem* for *MetFrag* tests were cached in a local database to exclude influences from network connectivity. Similarly, general spectral data required to post-process *FeatureFinderMetabo* results were cached, as this is usually loaded once during a workflow, even with varying input parameters. The input features for *GenForm* tests that resulted in very long individual run times (i.e. > 30 s) were removed to avoid excessive benchmark runtimes. Generating feature and MS peaklist input data for annotation related tests was performed with *patRoon* using algorithms from *OpenMS* and *mzR* [113], respectively. Pre-treatment of feature data consisted of removal of features with low intensity and lacking MS/MS data. The number of features for *SIRIUS* (except tests with native batch mode) and *MetFrag* benchmarks were further reduced by application of blank, replicate and intensity filters to avoid long total runtimes due to their relatively high individual run times. Finally, the feature dataset was split in low (0-500) and high (500-1000) *m/z* portions, which were purposed for execution of “simple” and “complex” experiments, respectively. For more details of the workflow and input parameters see the *R* script code in Additional file 4. The software tools used for benchmarking are summarized in Additional file 1.

**Table 4:** Utilized conditions for “simple” and “complex” tests

	Test	Input conditions^a^	Executions	Mean individual run time^b^ (s)
*msConvert*	Simple	Conversion centroided input	15	4.8
	Complex	Centroiding and conversion non-centroided input	15	8.5
*FeatureFinderMetabo*^c^	Simple	High intensity threshold	15	4.1
	Complex	Low intensity threshold	15	38
*GenForm*	Simple	CHNO elements, low *m/z*	512	0.2
	Complex	CHNOPS elements, high *m/z*	128	1.7
*SIRIUS*^*c*^	Simple	CHNO elements, low *m/z*	152 (512^d^)	2.3
	Complex	CHNOPS elements, high *m/z*	44 (128^d^)	7.7
*MetFrag*^*c*^	Simple	Limited scoring, narrow mass search (5 ppm), low *m/z*	152	3.0
	Complex	Thorough scoring, wide mass search (20 ppm), high *m/z*	44	8.6

^a^Features with *m/z* 0–500 (low) and *m/z* 500–1000 (high)

^b^Based on a test run without parallelization (n = 3)

^c^Supports (configurable) native multithreading

^d^Number of executions for native batch mode benchmarks

When multiprocessing was used all tests (except *GenForm*_simple_, discussed below) showed a clear downward trend in execution times (down to ~ 200–500%), and optimum conditions were generally reached when the number of parallel processes equaled the number of physical cores (six, see Fig. 5a). When algorithms are fully parallelized, execution times are expected to follow an inverse relationship with the number of parallel process (i.e. 1/n) and this was observed most closely with *msConvert*, whereas execution times for other tools show a less steep reduction. Furthermore, utilizing multiple threads per core (i.e. hyperthreading) did not reduce execution times further and even slowed down in some cases (e.g. *MetFrag*_complex_). These deviations in scalability were not investigated in detail. Since they were more noticeable under complex conditions, it is expected that this may be caused by (a) more involved post-processing results after each execution, which is currently not parallelized, and (b) increased memory usage, which may raise the overhead of context switches performed by the operating system. Nevertheless, the experiments performed here clearly show that the multiprocessing functionality of *patRoon* can significantly reduce execution times of various steps in an NTA workflow.

An exception, however, was the test performed with *GenForm*_simple_, which exhibited no significant change in execution times with multiprocessing (Fig. 5a). Due to the particularly small mean run times (0.2 s) of this test, it was hypothesized that the overhead of instantiating a new process from *R* (inherently not parallelized) dominated the overall run times. To mitigate this, a ‘batch mode’ was implemented, where such process initiation occurs from a command shell sub-process instead. Here, multiple commands are executed by the sub-process in series, and the desired degree of parallelization is then achieved by launching several of these sub-processes and evenly dividing commands amongst them. The maximum size of each series (or “batch size”) is configurable, and represents a balance between reduction of process initiation overhead and potential loss of effectively load balancing of, for instance, commands with highly deviating execution times. Next, various batch sizes were tested for *GenForm*, both with and without multiprocessing parallelization (Additional file 2: Figure S4). For *GenForm*_simple_, execution times clearly decreased with increasing batch sizes, however, no further reduction was observed with parallelism. In contrast, serial execution of *GenForm*_complex_ was not affected by varying batch size, whereas added parallelism reduced execution times for small batch sizes (≤ 8), but significantly increased such times for larger sizes. The results demonstrate that the typical short lived *GenForm* executions clearly benefit from batch mode. In addition, it is expected that by further increasing the batch size for *GenForm*_simple_, overall lifetimes of batch sub-processes may increase sufficiently to allow better utilization of parallelization. However, since *GenForm*_complex_ results for larger batch sizes clearly show possible performance degradation for more complex calculations (e.g. due to suboptimal load balancing), eight was considered as a ‘safe’ default which improves overall performance for both simple and complex calculation scenarios (Fig. 5b).

Utilizing native multithreading for *FeatureFinderMetabo*, *SIRIUS* (without native batch mode) and *MetFrag* yields only relatively small reductions in their execution times (Fig. 5b). Under optimum conditions (6-8 threads), the most significant drop was observed for *SIRIUS*_complex_ (~ 40%), followed by *FeatureFinderMetabo*_simple_, *FeatureFinderMetabo*_complex_ and *MetFrag*_complex_-C (~ 20%). These results suggest that native multithreading only yields partial parallelization, which primarily occurs with complex input conditions. Note that *SIRIUS* supports different linear programming solvers (*Gurobi* [114], *CPLEX* [115] and the default *GLPK* [116]), which may influence overall performance and parallelization [117]. Nevertheless, a comparison between these solvers did not reveal significant changes with our experimental conditions (Additional file 2: Figure S5). Combining the multiprocessing functionality with native multithreading under optimum conditions (i.e. 6 parallel processes/threads) only reduces execution times for *SIRIUS*_complex_ (Fig. 5b). As such, both performance improvements and scalability of the multiprocessing implementation of *patRoon* appear highly effective at this stage.

The native batch modes of *msConvert* and *SIRIUS* allow calculations from multiple inputs within a single execution. This reduces the total number of tool executions, which may (1) lower the accumulated overhead associated with starting and finishing tool executions and (2) hamper effective parallelization from multiprocessing, especially if executions are less than the available CPU cores. The combination of multiprocessing (optimum conditions) and native batch mode was benchmarked with increasing number of inputs per tool execution (i.e. the native batch size; Additional file 2: Figure S6). For *msConvert*, execution times were largely unaffected by the input batch size if multiprocessing was disabled, which indicates a low execution overhead. Lowest execution times were observed when multiprocessing was enabled with small batch sizes (≤ 25% of the total inputs), which indicates a lack of native parallelization support. In contrast, *SIRIUS* showed significantly lower overall execution times with increasing batch sizes (up to ~ 7000% and ~ 320% for *SIRIUS*_simple_ and *SIRIUS*_complex_, respectively), while enabling multiprocessing did not reduce execution times for batch sizes > 1. These results show that (1) *SIRIUS* has a relative large execution overhead, which impairs multiprocessing performance gains, and (2) supports effective native parallelized batch execution. Thus, *SIRIUS* performs most optimal if all calculations are performed within a single execution. Similar to previous *SIRIUS* benchmarks, no significant differences were found across different linear solvers (Additional file 2: Figure S7). The results demonstrate that multiprocessing may improve efficiency for batch calculations with tools with low execution overhead and/or lack of native parallelization. Nonetheless, the dramatic improvement in *SIRIUS* calculation times when using the native batch mode indicates that software authors should generally consider implementing native threaded batch mode functionality if large batch calculations are an expected use case.

Finally, the implemented optimization strategies were tested for a complete *patRoon* NTA workflow consisting of typical data processing steps and using all previously tested tools. The chosen input conditions roughly fell in between the aforementioned “simple” and “complex” conditions (see code in Additional file 4). Note that optimization strategies were unavailable for some steps (e.g. grouping of features and collection of MS peak lists), and native batch mode was not used in order to demonstrate the usefulness of multiprocessing for tools that do not support this (e.g. other tools than *msConvert* and *SIRIUS* and those potentially available in future versions of *patRoon*). Regardless, the benchmarks revealed a reduction in total run times of ~ 50% (from ~ 200 to ~ 100 min; Fig. 5c). Since execution times of each step may vary significantly, the inclusion of different combinations of steps may significantly influence overall execution times.

The use of multiprocessing for all tools (except *SIRIUS*), the implemented batch mode strategies for *GenForm* and the use of the native batch mode supported by *SIRIUS* were set as default in *patRoon* with the determined optimal parameters from the benchmarks results. However, the user can still freely configure all these options to potentially apply further optimizations or otherwise (partially) disable parallelization to conserve system resources acquired by *patRoon*.

As a final note, it is important to realize that a comparison of these benchmarks with standalone execution of investigated tools is difficult, since reported execution times here are also influenced by (a) preparing input and processing output and (b) other overhead such as process creation from *R*. However, (b) is probably of small importance, as was revealed by the highly scalable results of *msConvert* where the need to perform (a) is effectively absent. Furthermore, the overhead from (a) is largely unavoidable, and it is expected that handling of input and output data is still commonly performed from a data analysis environment such as *R*. Nonetheless, the various optimization strategies employed by *patRoon* minimize such overhead, and it was shown that the parallelization functionality often provide a clear advantage in efficiency when using typical CLI tools in an *R* based NTA workflow, especially considering the now widespread availability of computing systems with increasing numbers of cores.

### Demonstration: suspect screening

The previous section investigated several parallelization strategies implemented in *patRoon* for efficient data processing. A common method in environmental NTA studies to increase data processing efficiency and reducing the data complexity is by merely screening for chemicals of interest. This section demonstrates such a suspect screening workflow with *patRoon*, consisting of (a) raw data pre-treatment, (b) extracting, grouping and suspect screening of feature data, and finally (c) annotating features to confirm their identity. During the workflow several rule-based filters are applied to improve data quality. The ‘suspects’ in this demonstration are, in fact, a set of compounds spiked to influent samples (Table 5), therefore, this brief NTA primarily serves for demonstration purposes. After completion of the suspect screening workflow, several methods are demonstrated to inspect the resulting data.

**Table 5:** Spiked compounds and their annotation rankings obtained with the demonstrated suspect screening workflow

Spiked compound	Spike concentration (µg/l)	Retention time^a^ (min)	*m/z*^a^	Compound rank	Formula rank
(4/5)-Methylbenzotriazole^b^	1	10.0/10.1	134.0709	2/4	1
Aniline	1	–	–	–	–
Barbital	10	2.3	185.0918	1	1
Benzotriazole	1	8.0	120.0553	1	1
Carbamazepine	1	13.3	237.1018	1	2
Carbendazim	1	6.3	192.0764	1	1
Dimethomorph^c^	1	16.2/16.6	388.1303	1/1	25/21
Gabapentin	1	6.4	172.1328	1	1
Hexamethylenetetramine	3	2.1	141.1132	1	1
Melamine^c^	3	2.1/2.3	127.0724	1/1	1/1
Metformin	5	2.2	130.1084	1	1
Propranolol	1	11.8	260.1640	1	1
Terbuthylazine	1	16.9	230.1163	1	2
Tetraglyme	3	7.8	223.1536	1	1
Tiamulin	1	13.8	494.3290	1	3
Tramadol	1	9.4	264.1953	1	1
Triphenylphosphine oxide	1	15.4	279.0928	1	2

^a^Averaged value from feature group assigned to suspect

^b^A mixture was spiked (35%/65%), experimental retention times were not determined and therefore unknown

^c^Two chromatographic peaks observed [11]

### Suspect screening: workflow

The code described here can easily be generated with the newProject() function, which automatically generates a ready-to-use R script based on user input (section “Visualization, reporting and graphical interface”).

First, the *patRoon R* package is loaded and a data.frame is generated with the file information of the sample analyses and their replicate and blank assignments. Next, this information is used to centroid and convert the raw analyses files to the open mzML file format, a necessary step for further processing.


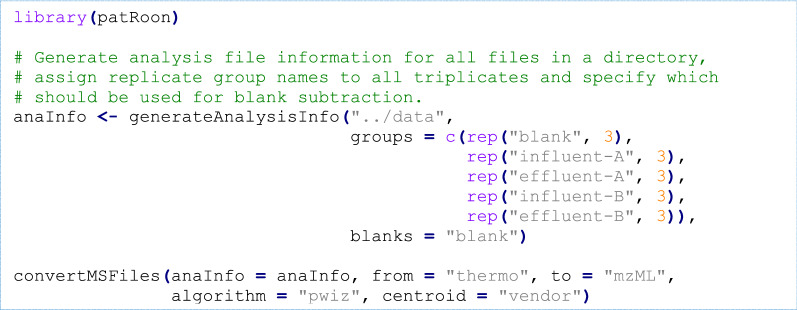


The next step involves finding features and grouping them across samples. This example uses the *OpenMS* algorithms and sets several algorithm specific parameters that were manually optimized for the employed analytical instrumentation to optimize the workflow output. Other algorithms (e.g. *enviPick*, *XCMS*) are easily selected by changing the algorithm function parameter.


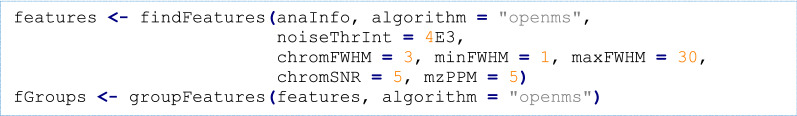


Several rule-based filters are then applied for general data clean-up, followed by the removal of sample blanks from the feature dataset.


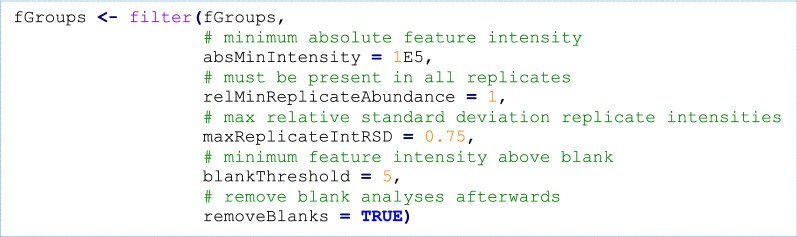


Next, features are screened with a given suspect list, which is a CSV file read into a data.frame containing the name, SMILES and (optionally) retention time for each suspect (see Additional file 5). While the list in this demonstration is rather small (18 compounds, see Table 5), larger lists containing several thousands of compounds such as those available on the NORMAN network Suspect List Exchange [118] can also be used. The screening results are returned in a data.frame, where each row is a hit (a suspect may occur multiple times) containing the linked feature group identifier and other information such as detected *m/z* and retention time (deviations). Finally, this table is used to transform the original feature groups object (fGroups) by removing any unassigned features and tagging remainders by their suspect name.


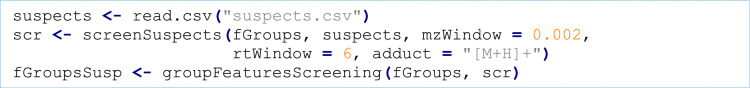


In the final step of this workflow annotation is performed, which consists of (a) generation of MS peak list data, (b) general clean-up to only retain significant MS/MS mass peaks, automatic annotation of (c) formulae and (d) chemical compounds, and (e) combining both annotation data to improve ranking of candidate compounds. As with previous workflow steps, the desired algorithms (*mzR*, *GenForm* and *MetFrag* in this example) are set using the algorithm function parameter. Similarly, the compound database used by *MetFrag* (here *CompTox* via a local CSV file obtained from [119]) can easily be changed to other databases such as *PubChem*, *ChemSpider* or another local file.


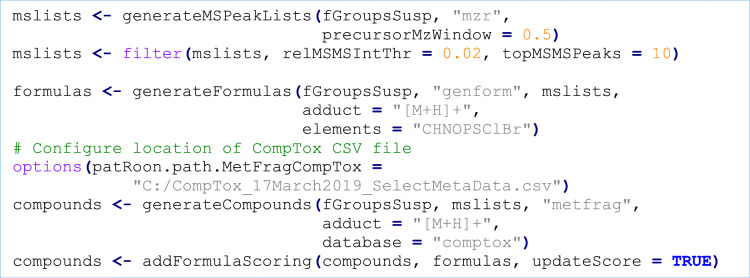


### Suspect screening: data inspection

All data generated during the workflow (e.g. features, peak lists, annotations) can be inspected by overloads of common *R* methods.


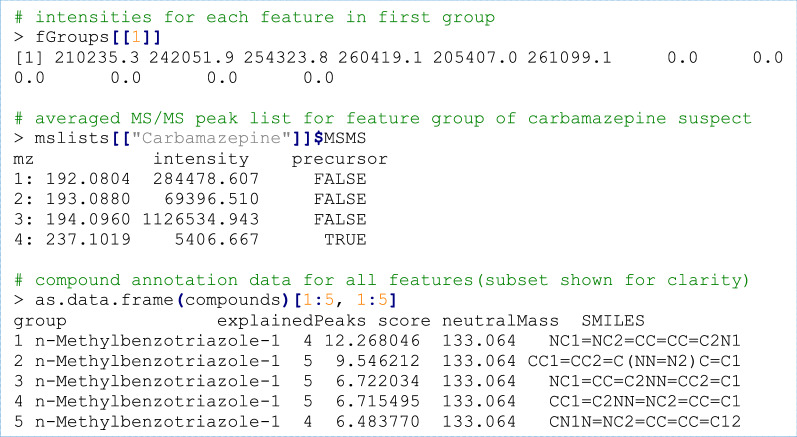


Furthermore, all workflow data can easily be subset with, e.g. the *R* subset operator (“[“), for instance, to perform a (hypothetical) prioritization of features that are most intense in the effluent samples.


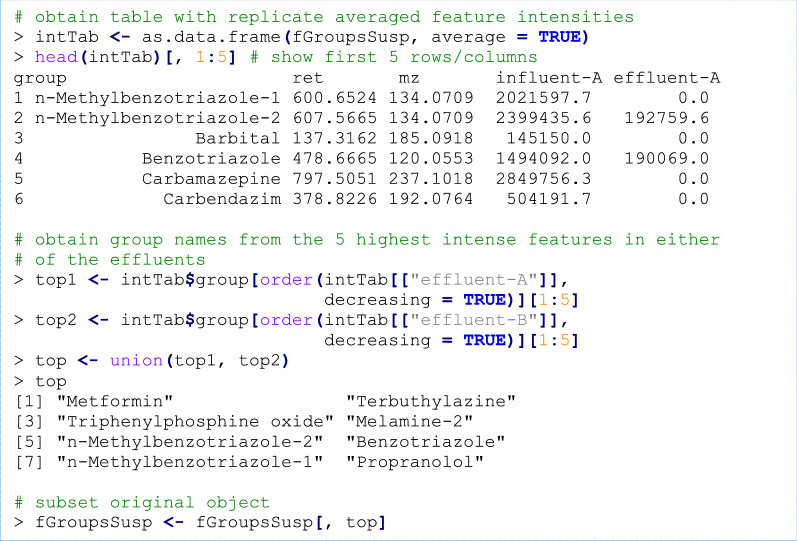


**Fig. 6:**
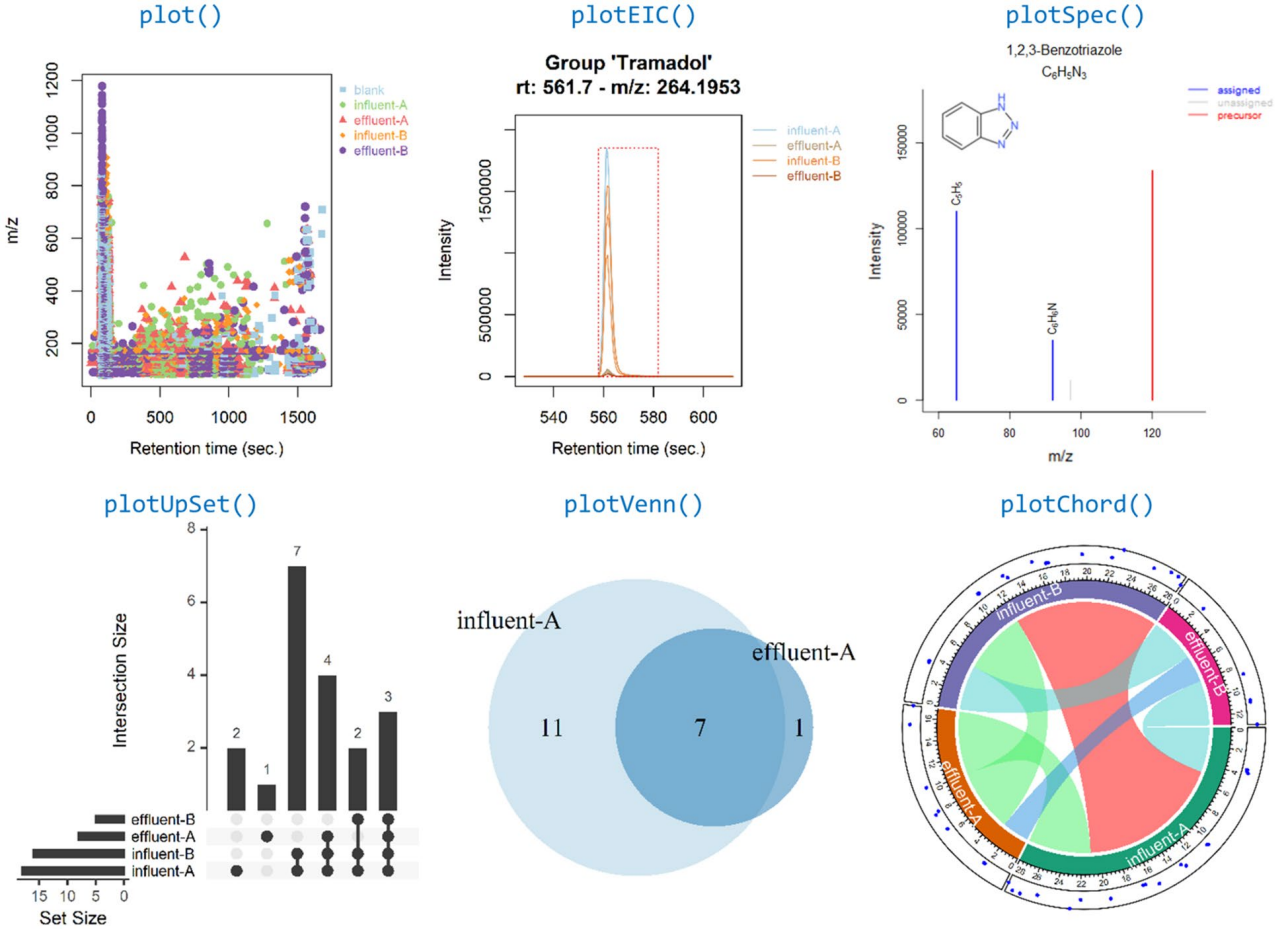
Common visualization functionality of *patRoon* applied to the demonstrated workflow. From left to right: an *m/z vs* retention time plot of all feature groups uniquely present in the samples, an EIC for the tramadol suspect, a compound annotated spectrum for the benzotriazole suspect and comparison of feature presence between sample groups using UpSet [77], Venn (influent/effluent A) and chord diagrams


Visualization of data generated during the workflow, such as an overview of features, chromatograms, annotated MS spectra and uniqueness and overlap of features, can be performed by various plotting functions (see Fig. 6).

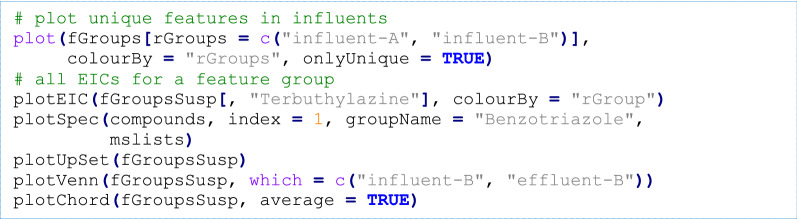


The final step in a *patRoon* NTA workflow involves automatic generation of comprehensive reports of various formats which allow (interactive) exploration of all data (see Additional file 2: Figure S8).


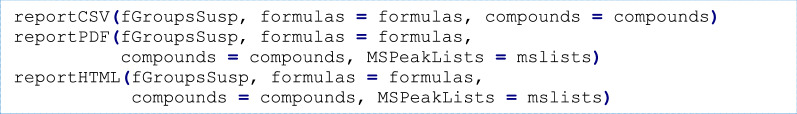


## Suspect screening: results

A summary of data generated during the NTA workflow demonstrated here is shown in Tables 5 and 6. The complete workflow finished in approximately 8 min (employing a laptop with an Intel^®^ Core™ I7-8550U CPU, 16 gigabyte RAM, NVME SSD and the Windows 10 Pro operating system). While nearly 60,000 features were grouped into nearly 20,000 feature groups, the majority (97%, 678 remaining) were filtered out during the various pre-treatment filter steps. Regardless, most suspects were found (17/18 attributed to 19/20 individual chromatographic peaks, Table 5), and the missing suspect (aniline) could be detected when lowering the intensity threshold of the filter() function used to post-filter feature groups in the workflow. The majority of suspects (17) were annotated with the correct chemical compound as first candidate (Table 6), the two n-methylbenzotriazole isomer suspects were ranked as second or fourth. Results for formulae assignments were similar, with the exception of dimethomorph, where the formula was ranked in only the top 25 (the candidate chemical compound was ranked first, however).

**Table 6:** Summarizing results for the demonstrated patRoon NTA workflow

		Amount
Features	Total found	57,113 (mean 3808/sample)
Feature groups	Raw dataset	19,970
	Replicate filters (1st pass^a^)	4719 (− 76%)
	Blank filter	2933 (− 85%)
	Intensity filters	964 (− 95%)
	Replicate filters (2nd pass^a^)	678 (− 97%)
Suspects	Total found	19 out of 20
	Annotated	19
Formulae	Total candidates	163 (mean 9/feature group)
	Correctly ranked 1st	13 (68%)
	Correctly ranked 1st–2nd	16 (84%)
	Correctly ranked 1st–5th	17 (89%)
Compounds	Total candidates	1017 (mean 54/feature group)
	Correctly ranked 1st	17 (85%)
	Correctly ranked 1st–2nd	18 (90%)
	Correctly ranked 1st–5th	19 (100%)

^a^Replicate filters are repeated if necessary, see section “Data reduction, comparison and conversion”

While this demonstration conveys a relative simple NTA with ‘known suspects’, the results show that *patRoon* is (a) time-efficient on conventional computer hardware, (b) allows a straightforward approach to perform a complete and tailored NTA workflow, (c) provides powerful general data clean-up functionality to prioritize data and (d) performs effective automated annotation of detected features.

### Demonstration: algorithm consensus

This section briefly demonstrates how the consensus functionality of *patRoon* can be used to compare and combine output from the supported algorithms from *OpenMS*, *XCMS* and *enviPick*. The MS data from the suspect screening demonstration above was also used here. The full processing script can be found as Additional file 6.

To obtain the feature data the findFeatures(), groupFeatures() and filter() functions were used as was demonstrated previously (see Additional file 6). The first step is to create a comparison from this data, which is then used to create a consensus (discussed in section “Data reduction, comparison and conversion”). The consensus can be formed from combining all data or from overlapping or unique data, which can then be inspected with the aforementioned data inspection functionality.


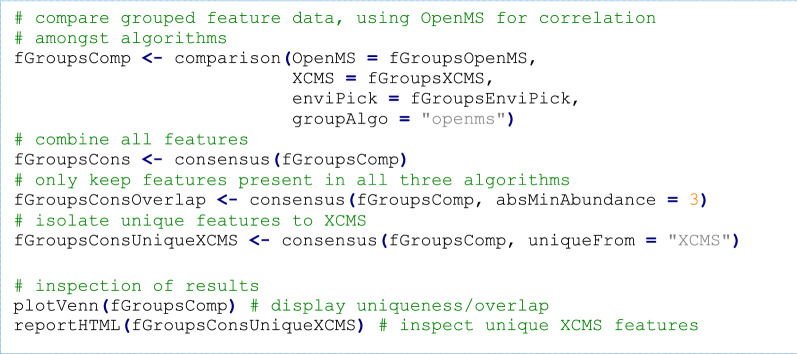


A summary of the results is shown in Table 7 and Additional file 2: Figure S9. While the number of features prior to grouping and filtering varied significantly between algorithms (~ 10 000 to ~ 60 000), they were roughly equal after pre-treatment: 678 (*OpenMS*), 801 (*XCMS*) and 836 (*enviPick*). Combining these resulted in 1243 grouped features, of which 541 (44%) were unique to one algorithm, 332 (27%) were shared amongst two algorithms and 370 (30%) fully overlapped. Application of the suspect screening workflow from the previous section revealed that the same 17 out of 18 suspects were present in all the algorithm specific, combined and overlapping feature datasets. Still, the results from this demonstration indicates that each algorithm generates unique results. Dedicated efforts such as ENTACT [120–122] will help to unravel the importance of unique and overlapping algorithm results, however, such studies are out of the scope of this article. Regardless, this demonstration showed how *patRoon* provides researchers the tools needed to easily use and combine workflow data from different algorithms to perform such an evaluation for their use cases.

**Table 7:** Summary of the feature consensus demonstration results. Workflow details can be found in Additional file 6

	Algorithm^a^			Consensus	
	*OpenMS*	*XCMS*	*enviPick*	Combined	Full overlap
Features	57,113	32,078	11,431		
Feature groups (un-filtered)	19,970	11,166	2809		
Feature groups	678 (*95*)	801 (*238*)	836 (*208*)	1243	370
With formulas	521 (*75*)	614 (*169*)	656 (*168*)	955	291
With compounds^b^	251 (*33*)	291 (*68*)	298 (*62*)	440	159
Detected suspects	17 of 18	17 of 18	17 of 18	17 of 18	17 of 18

^a^Italic values in parentheses are unique to the algorithm

^b^Using the EPA CompTox database

## Conclusions

This paper presents *patRoon*, a fully open source platform that provides a comprehensive MS based NTA data processing workflow developed in the *R* environment. Major workflow functionality is implemented through the usage of existing and well-tested software tools, connecting primarily open and a few closed approaches. The workflows are easily setup for common use cases, while full customization and mixing of algorithms allows for execution of completely tailored workflows. In addition, extensive functionality related to data processing, annotation, visualization, reporting and others was implemented in *patRoon* to provide an important toolbox for effectively handling complex NTA studies. The easy and predictable interface of *patRoon* lowers the computational expertise required of users, making it available for a broad audience. It was shown that the optimization strategies implemented reduced the computational times. Furthermore, it was demonstrated how *patRoon* can be used to perform a straightforward and effective suspect screening workflow and how it can easily generate, compare and combine results from different NTA workflow algorithms.

*patRoon* has been under development for several years and has already been applied in a variety of studies, such as the characterization of organic matter [71], elucidation of transformation products of biocides [7, 12], assessment of removal of polar organics by reversed-osmosis drinking water treatment [14] and the investigation of endocrine disrupting chemicals in human breast milk [110]. *patRoon* will be maintained to stay compatible with its various dependencies and further development is planned. This includes extension of integrated workflow algorithms for new and less commonly used ones and the implementation of additional componentization strategies to help prioritizing data. Addition of new workflow functionality is foreseen, such as usage of ion-mobility spectrometry data to assist annotation, automated screening of transformation products (e.g. utilizing tools such as *BioTransformer* [123]), prediction of feature quantities for prioritization purposes (recently reviewed in [124]) and automated chemical classification (e.g. through *ClassyFire* [125]). Finally, interfacing with other *R* based mass spectrometry software such as those provided by the “R for Mass Spectrometry” initiative [126] is planned to further improve the interoperability of *patRoon*. The use in real-world studies, feedback from users and developments within the non-target analysis community, are all critical in determining future directions and improvements of *patRoon*. We envisage that the open availability, straightforward usage, vendor independence and comprehensive functionality will be useful to the community and result in a broad adoption of *patRoon*.

### Availability and requirements

Project name: patRoon.

Project home page: https://github.com/rickhelmus/patRoon.

Operating system(s): Platform independent (tested on Microsoft Windows and Linux).

Programming language(s): R, C ++, JavaScript.

Other requirements: Depending on utilized algorithms (see installation instructions in [85, 88]).

License: GNU GPL version 3.

Any restrictions to use by non-academics: none.

### Definitions

Features: data points assigned with unique chromatographic and mass spectral information (e.g. retention time, peak area and accurate m/z), which potentially described a compound in a sample analysis.

Feature group: A group of features considered equivalent across sample analyses.

MS peak list: tabular data (*m/z* and intensity) for MS or MS/MS peaks attributed to a feature and used as input data for annotation purposes.

Formula/Compound: a chemical formula or compound candidate revealed during feature annotation.

Component: A collection of feature groups that are somehow linked, such as MS adducts, homologous series or highly similar intensity trends.

## Supplementary information

**Additional file 1.** Overview of software and databases that are used in the implementation in *patRoon*. This table summarizes all the software and databases that are described in the implementation section of the main text.

**Additional file 2.** Additional figures that illustrate implementation details of *patRoon* and miscellaneous benchmarking and demonstration results.

**Additional file 3.** Additional tables with more details on the implementation.

**Additional file 4.** Source code for benchmarks. Archive with several *R* scripts that were used to perform the parallelization benchmarks.

**Additional file 5.** Demonstration suspect list. Suspect list that was used for the *patRoon* demonstration. The list was based on the detected compounds reported in [11], and SMILES identifiers for each suspect were collected from PubChem [24].

**Additional file 6.** Algorithm consensus demonstration. Script that was used to generate the results for the feature algorithm consensus demonstration.

## Data Availability

The source code of *patRoon* and online versions of its manuals are available for download from https://github.com/rickhelmus/patRoon and archived in [85, 127]. The raw data used for benchmarking and demonstration purposes in this manuscript is archived in [111]. The scripts used to perform benchmarking and the input suspect list for demonstration purposes are provided as Additional file 4 and 5, respectively.

## Abbreviations

CECs: Contaminants of emerging concern
CLI: Command-line interface
CSV: Comma-separated value
DBI: The database interface
EIC: Extracted ion chromatogram
GC: Gas chromatography
GC-MS: GC coupled to mass spectrometry
HTML: Hypertext markup language
HRMS: High resolution mass spectrometry
IPO: Isotopologue parameter optimization
LC: Liquid chromatography
LC–MS: LC coupled to mass spectrometry
MS/MS: Tandem mass spectrometry
NTA: Non-target analysis
PDF: Portable document format
XCMS: Various forms (X) of chromatography mass spectrometry (*R* package MS data processing)
