# Supplementary material for: patRoon: open source software platform for environmental mass spectrometry based non-target screening
Source: J Cheminform. 2021 Jan 6;13:1. doi: 10.1186/s13321-020-00477-w (PMC7789171; doi:10.1186/s13321-020-00477-w)
Supplement: Supplementary file 2 — Additional file 2. Additional figures that illustrate implementation details of patRoon and miscellaneous benchmarking and demonstration results. [file 13321_2020_477_MOESM2_ESM.docx]

# patRoon: Open source software platform for environmental mass spectrometry based non-target screening

Rick Helmus^a*^, Thomas L. ter Laak^a,b^, Annemarie P. van Wezel^a^, Pim de Voogt^a^ and Emma L. Schymanski^c^

^a^ Institute for Biodiversity and Ecosystem Dynamics, University of Amsterdam, P.O. Box 94240, 1090 GE Amsterdam, The Netherlands
^b^ KWR Water Research Institute, Chemical Water Quality and Health, P.O. Box 1072, 3430 BB Nieuwegein, The Netherlands
^c^ Luxembourg Centre for Systems Biomedicine (LCSB), University of Luxembourg, L-4367 Belvaux, Luxembourg.

* Corresponding author: r.helmus@uva.nl

# Supplementary information


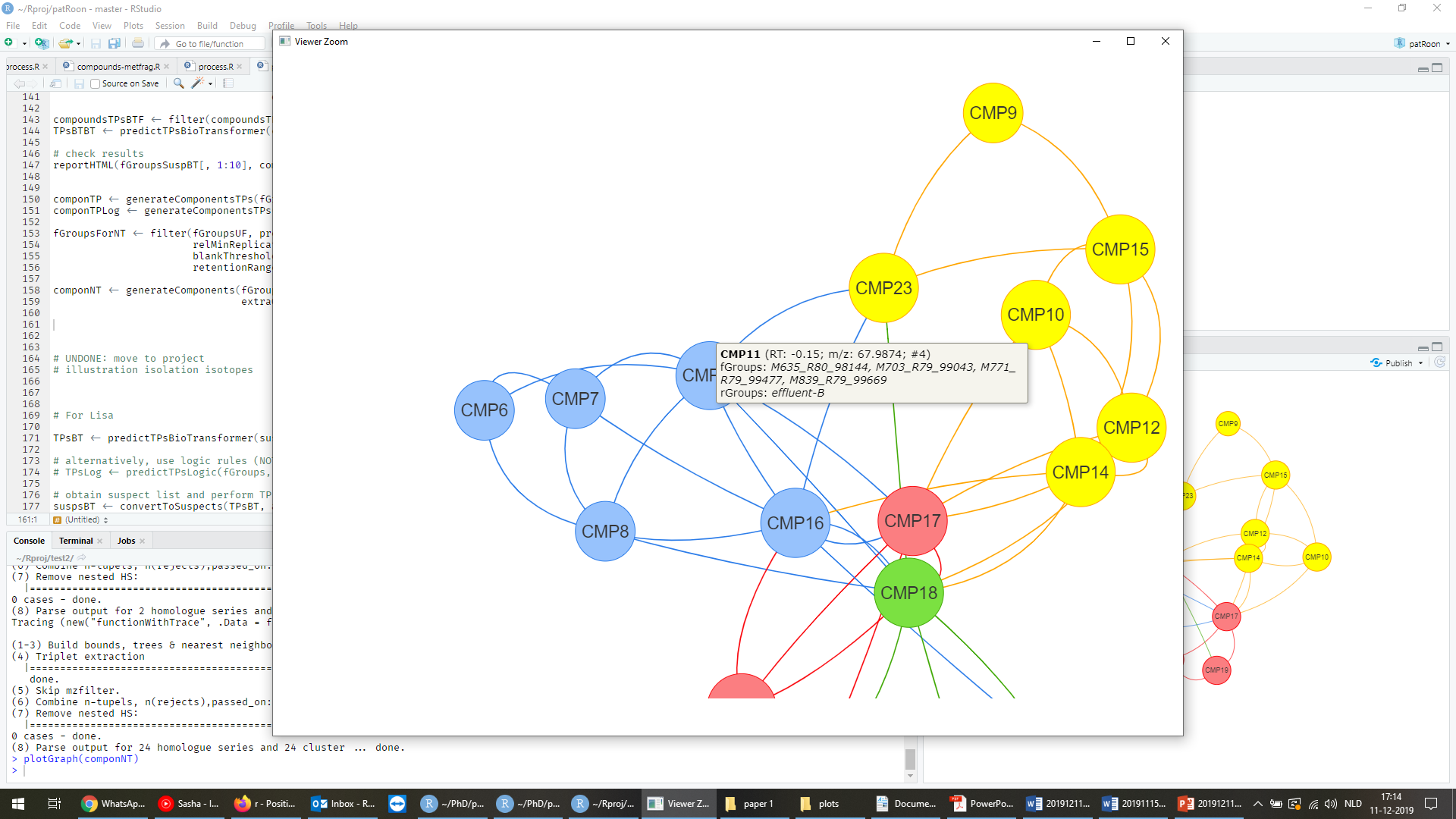


Figure S1. Componentization (CMT) of features based on their participation in homologous series may yield components with overlapping features, which cannot be merged without resulting in ambiguities to other series. These components and their links can be explored by an interactive network graph. The different color codes represent dense subgraphs, i.e. series that show large overlap in features.


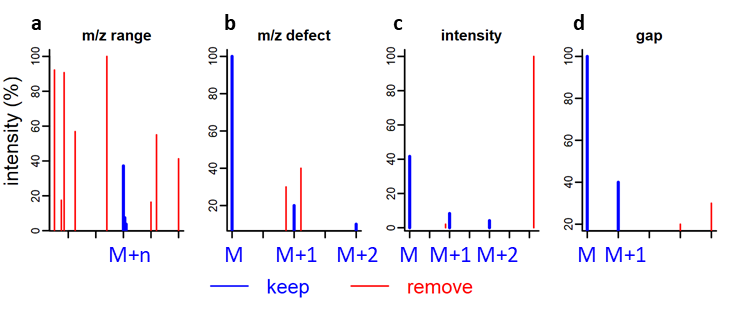


Figure S2. Filters to isolate isotopic feature masses from MS data. The feature mass is assumed to be monoisotopic (“M”), and isotopic peaks are recognized if (a) “m/z range”: the m/z is between that of the feature and an expected maximum isotopic cluster size, (b) “m/z defect”: the mass defect is close to that of the M (the tolerance propagates with feature distance), (c) “intensity”: the intensity compared to the feature falls within an expected range and (d) “gap”: no ‘gap’ exists between detection of M+n and M+(n+1).


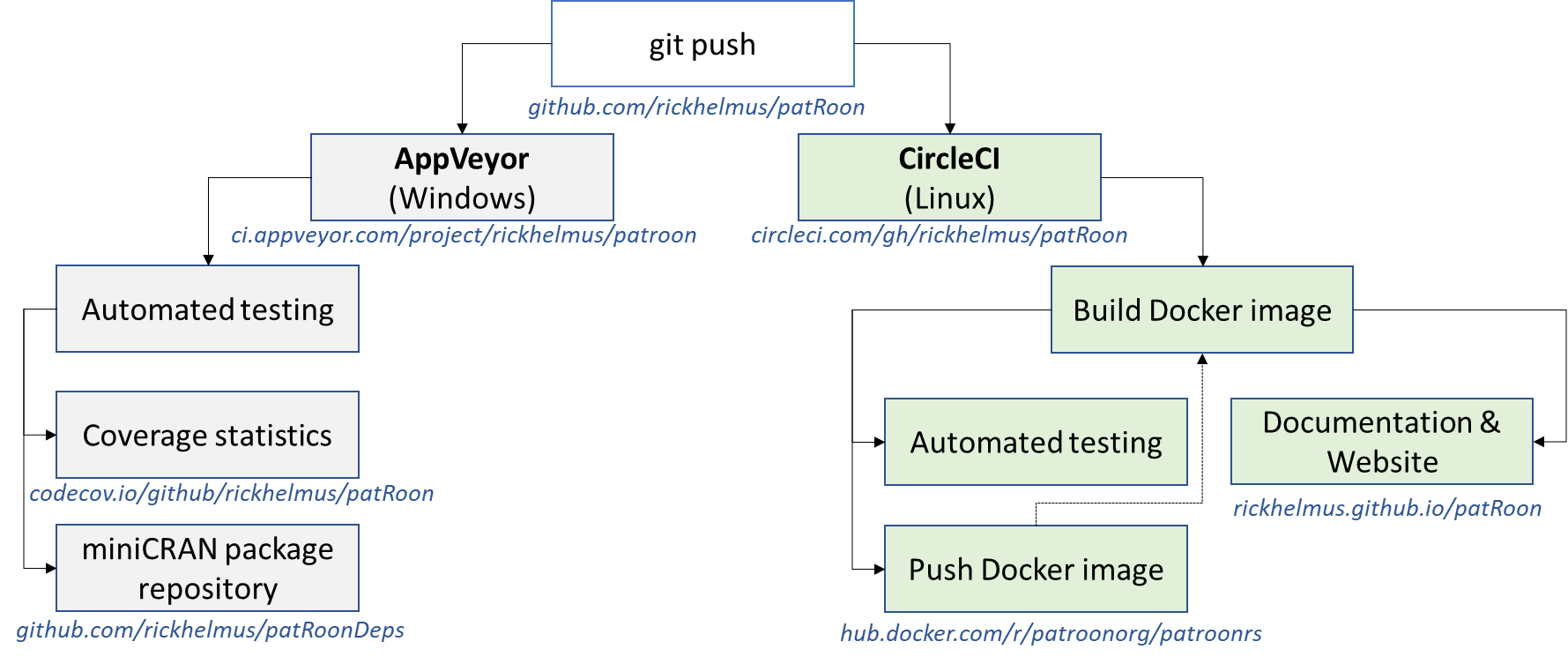


Figure S3. Continuous integration (CI) pipeline providing automated testing on Windows and Linux (hosted on AppVeyor [1] and CircleCI [2], respectively). The Windows CI platform publishes coverage statistics on Codecov [3] (using the covr R package [4]) and maintains a miniCRAN [5] R package repository containing patRoon and all its R dependencies. The Linux CI platform builds a Docker container with patRoon and its dependencies, which is subsequently used to perform automated testing and updating the online documentation and website in parallel. After completion of Linux tests, the Docker container is pushed to a repository for debugging purposes and re-used for building the container for the next test cycle. The Docker container contains RStudio [6], which makes it also highly suitable to easily obtain a full data processing environment with patRoon.


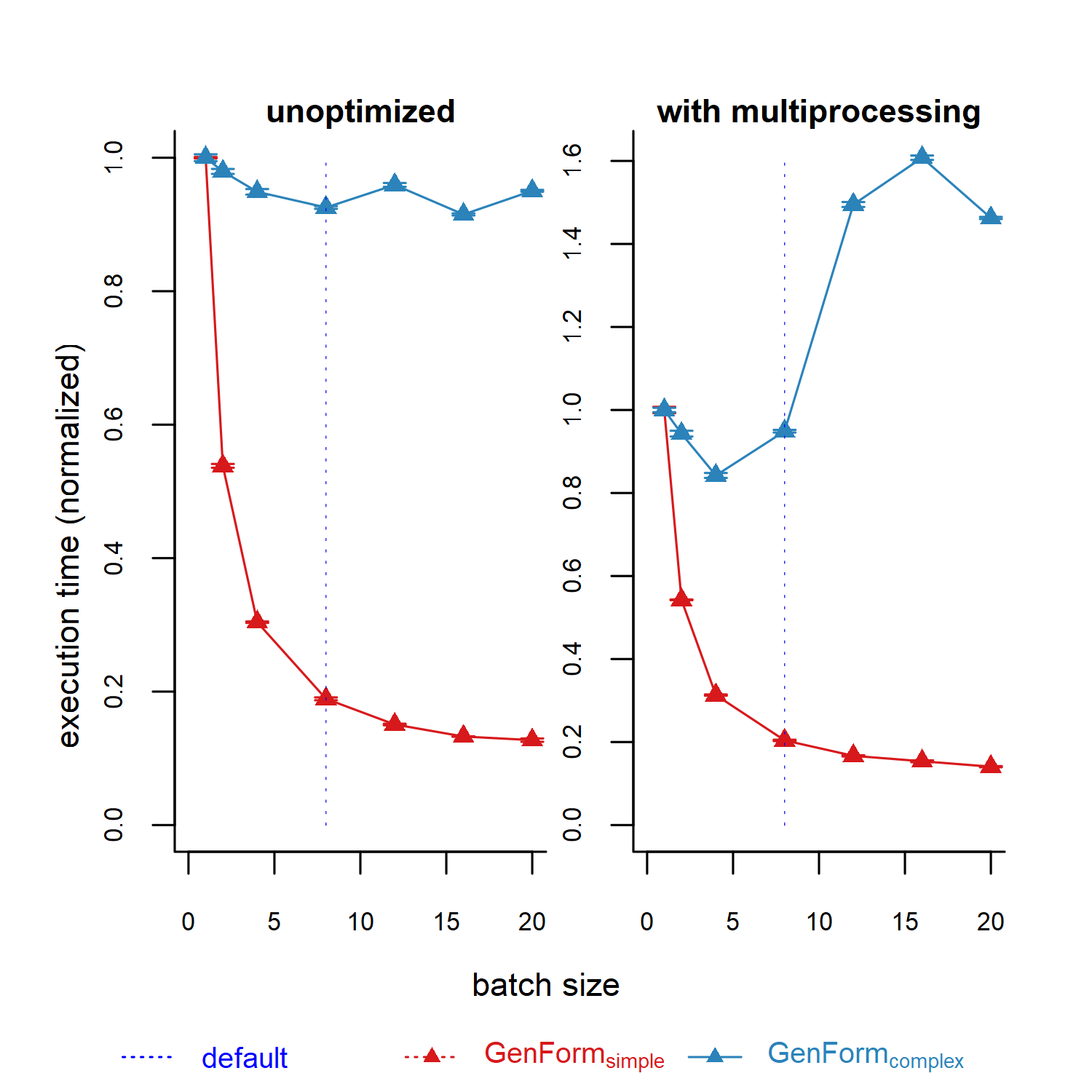


Figure S4.Batch mode benchmarks for GenForm with “simple” and “complex” conditions (see Table 4 in main text). The benchmarks were executed without (left) and with (right) multiprocessing enabled. Graphs represent the batch size versus relative execution time (normalized to batch size of one, n=3).


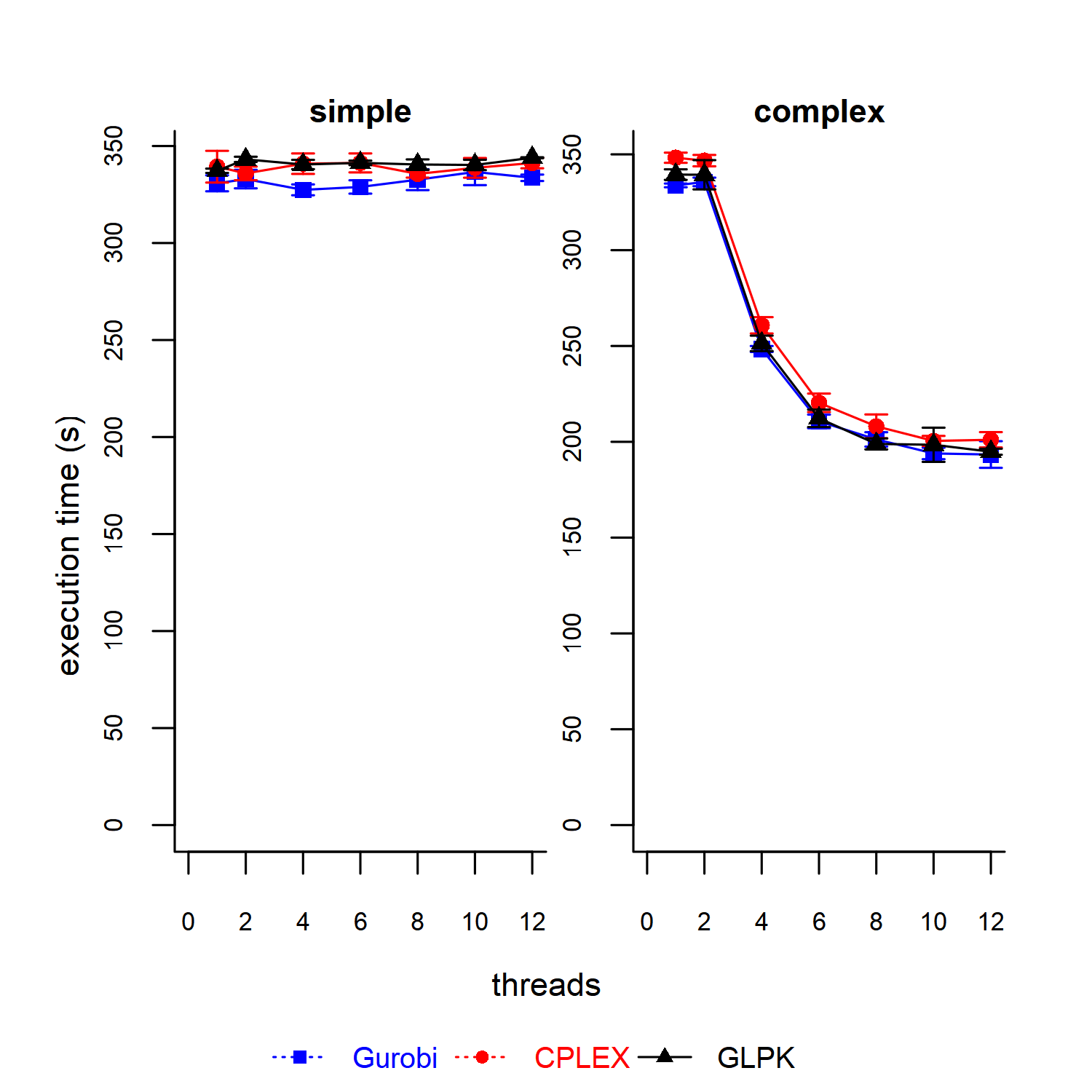


Figure S5.Parallelization benchmarks comparing SIRIUS with different linear solvers (Gurobi [7], CPLEX [8] and the default GLPK [9]) running with “simple” (left) and “complex” (right) input conditions (see Table 4 in main text). Graphs represent the number of native threads versus average execution time (n=3).


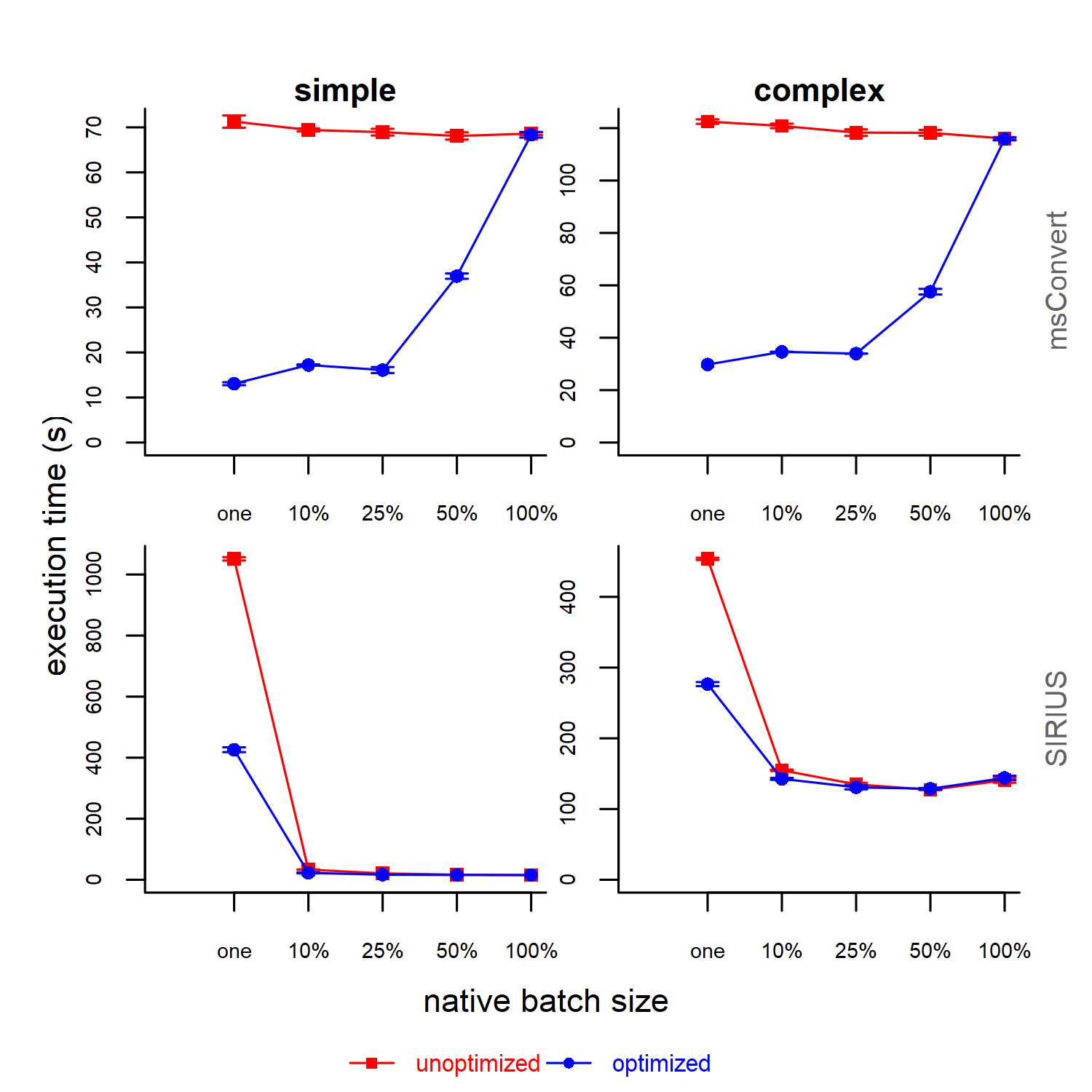


Figure S6.Benchmarks of the native batch modes of msConvert and SIRIUS with (blue) and without (red) multiprocessing enabled and tested on “simple” (left) and “complex” (right) input conditions (see Table 4 in main text) . Graphs represent the total execution time versus tested batch sizes (n=3), where the latter is either one or a fraction of the total amount of inputs.


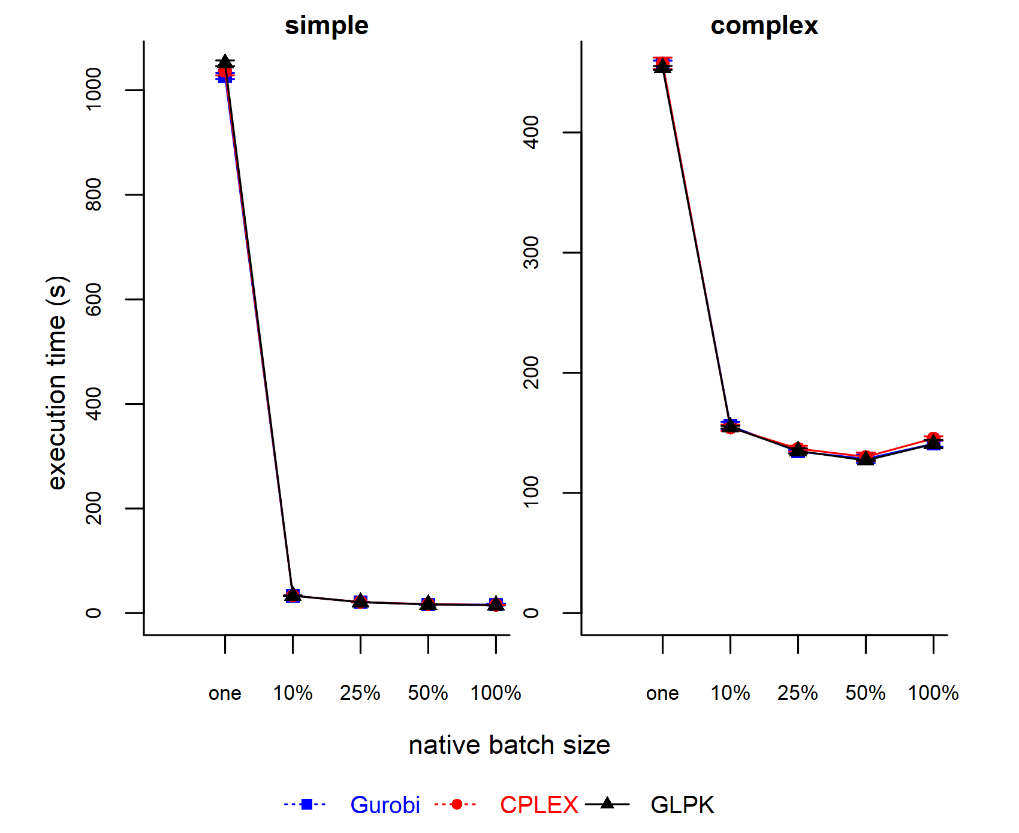


Figure S7. Benchmarks comparing native batch mode of SIRIUS with different linear solvers (Gurobi [7], CPLEX [8] and the default GLPK [9]) running with “simple” (left) and “complex” (right) input conditions (see Table 4 in main text). Graphs represent the total execution time versus tested batch sizes (n=3), where the latter is either one or a fraction of the total amount of inputs.


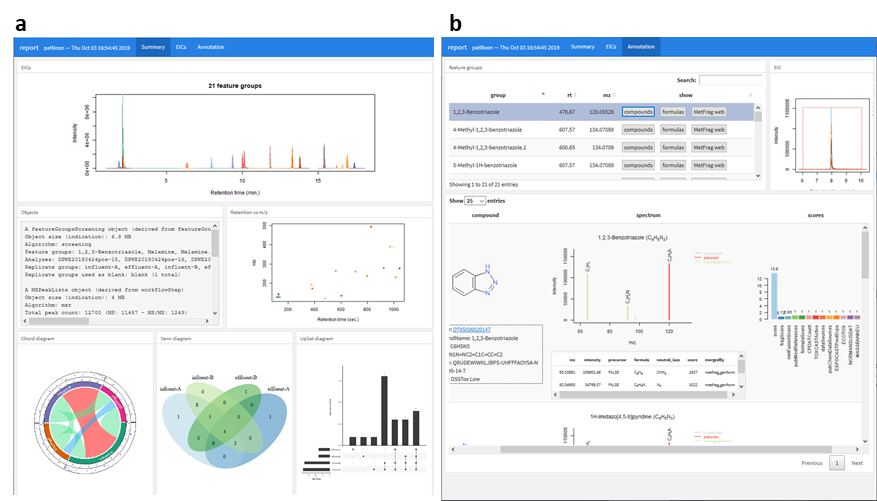


Figure S8. Interactive reporting functionality of patRoon showing an overview of all results (a) and annotation results (b) of the demonstrated NTA.


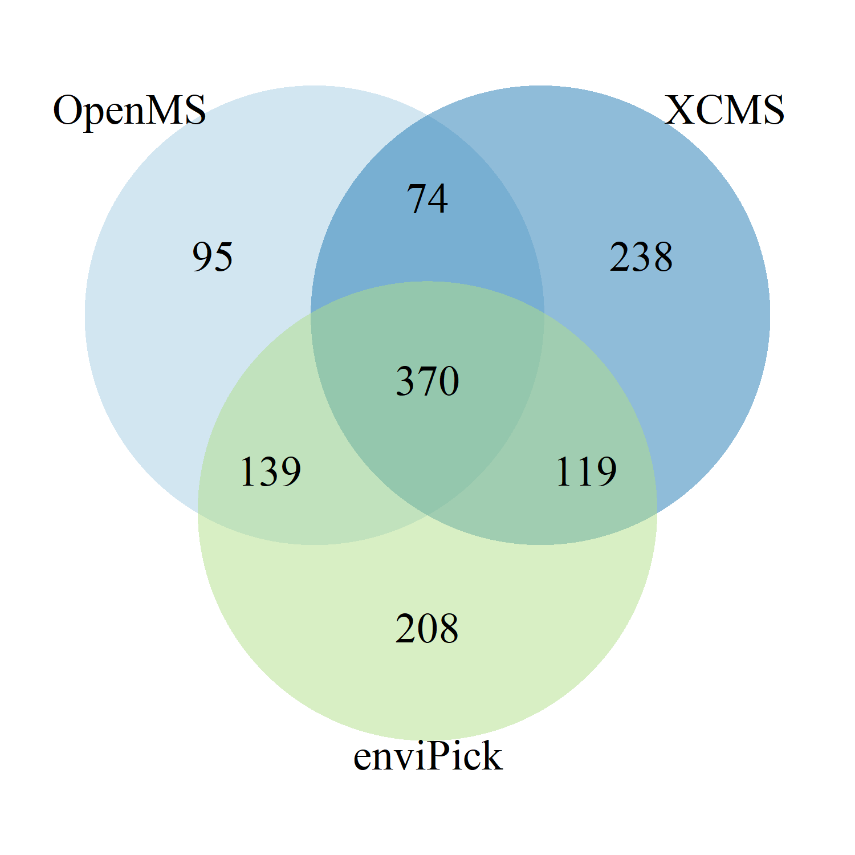


Figure S9.Comparison between resulting features from OpenMS, XCMS and enviPick of the algorithm consensus demonstration.

# References

1. AppVeyor: Continuous Integration and Deployment service for Windows, Linux and macOS. In: AppVeyor. https://www.appveyor.com/. Accessed 6 Feb 2020

2. CircleCI: Continuous Integration and Delivery. In: CircleCI. https://circleci.com/. Accessed 6 Feb 2020

3. Code Coverage Done Right. In: Codecov. https://codecov.io. Accessed 6 Feb 2020

4. Hester J (2019) covr: Test Coverage for Packages. https://CRAN.R-project.org/package=covr

5. Vries A de (2019) miniCRAN: Create a Mini Version of CRAN Containing Only Selected Packages. https://CRAN.R-project.org/package=miniCRAN

6. RStudio | Open source & professional software for data science teams. https://rstudio.com/. Accessed 19 Oct 2020

7. Gurobi. https://www.gurobi.com/. Accessed 6 Feb 2020

8. CPLEX Optimizer. https://www.ibm.com/analytics/cplex-optimizer. Accessed 6 Feb 2020

9. GNU Project - Free Software Foundation (FSF) GLPK (GNU Linear Programming Kit). https://www.gnu.org/software/glpk/. Accessed 6 Feb 2020
