# Supplementary material for: patRoon: open source software platform for environmental mass spectrometry based non-target screening
Source: J Cheminform. 2021 Jan 6;13:1. doi: 10.1186/s13321-020-00477-w (PMC7789171; doi:10.1186/s13321-020-00477-w)
Supplement: Supplementary file 3 — Additional file 3. Additional tables with more details on the implementation. [file 13321_2020_477_MOESM3_ESM.docx]

# patRoon: Open source software platform for environmental mass spectrometry based non-target screening

Rick Helmus^a*^, Thomas L. ter Laak^a,b^, Annemarie P. van Wezel^a^, Pim de Voogt^a^ and Emma L. Schymanski^c^

^a^ Institute for Biodiversity and Ecosystem Dynamics, University of Amsterdam, P.O. Box 94240, 1090 GE Amsterdam, The Netherlands
^b^ KWR Water Research Institute, Chemical Water Quality and Health, P.O. Box 1072, 3430 BB Nieuwegein, The Netherlands
^c^ Luxembourg Centre for Systems Biomedicine (LCSB), University of Luxembourg, L-4367 Belvaux, Luxembourg.

* Corresponding author: r.helmus@uva.nl

# Supplementary information

Table S1. Software tools integrated in patRoon that implement most of the workflow functionality.

|  | **HRMS data** | **Features** | | | **Annotation** | | | **Processing** |
| --- | --- | --- | --- | --- | --- | --- | --- | --- |
|  | **PT** | **PO** | **FTS** | **FG** | **MSPL** | **FOR** | **COM** | **CMT** |
| *ProteoWizard* | X^1^ |  |  |  |  |  |  |  |
| *IPO*^2^ |  | X |  |  |  |  |  |  |
| *OpenMS* | X^3^ |  | X^4^ | X^5^ |  |  |  |  |
| *XCMS* |  |  | X | X |  |  |  |  |
| *enviPick* |  |  | X |  |  |  |  |  |
| *DataAnalysis* | X |  | X |  | X | X^6^ |  |  |
| *ProfileAnalysis* |  |  |  | X^6^ |  |  |  |  |
| *TASQ* |  |  |  | X^7^ |  |  |  |  |
| *mzR* |  |  |  |  | X |  |  |  |
| *GenForm* |  |  |  |  |  | X |  |  |
| *SIRIUS* |  |  |  |  |  | X | X |  |
| *MetFrag* |  |  |  |  |  |  | X |  |
| *CAMERA* |  |  |  |  |  |  |  | X |
| *RAMClustR* |  |  |  |  |  |  |  | X |
| *nontarget* |  |  |  |  |  |  |  | X |
| PT: data pre-treatment; PO: feature parameter optimization; FTS: features; FG: feature groups; MSPL: MS peak lists; FOR: formulae; COM: compounds; CMT: components; (1): using msConvert; (2) customized code base, see main text; (3) using *FileConverter*; (4): using *FeatureFinderMetabo*; (5): using *MapAlignerPoseClustering* (retention time alignment) and *FeatureLinkerUnlabeled*(*QT*) (grouping); (6): requires features from DataAnalysis as input; (7): conversion of screening results to grouped features. | | | | | | | | |
